# Antioxidant strategies against cellular senescence: unveiling the power of synthetic versus natural antioxidants in a systematic review

**DOI:** 10.3389/fragi.2025.1543360

**Published:** 2025-05-27

**Authors:** Farbod Ebrahimirad, Seyedeh Elahe Mirmahdizade, Bahar Mahmoodieh, Saina Najafi, Seyedeh Marzieh Banihashemian, Sadegh Nikakhtar, Hesam Mobaraki, Asma Sadeghi, Naghmeh Kossari, Seyyed Kiarash SadatRafiei, Masood Ghodsi Moghadam, Ali Mashkani, Mahsa Asadi Anar, Farbod khosravi

**Affiliations:** ^1^ Student Research Committee, Shahid Beheshti University of Medical Sciences, Tehran, Iran; ^2^ Department of Food Science and Technology, Science and Research Branch, Islamic Azad University, Tehran, Iran; ^3^ School of Medicine, Iran University of Medical Sciences, Tehran, Iran; ^4^ School of Advanced Sciences and Technology, Tehran Medical Branch, Islamic Azad University, Tehran, Iran; ^5^ Department of Clinical Biochemistry, Faculty of Medical Sciences, Tarbiat Modares University, Tehran, Iran; ^6^ Faculty of Medicine, İstanbul Yeniyuzyil University, İstanbul, Türkiye; ^7^ Department of Food Science and Technology, Faculty of Agriculture, University of Tabriz, Tabriz, Iran; ^8^ Student Research Committee, University of Social Welfare and Rehabilitation Sciences, Tehran, Iran; ^9^ School of Medicine, Shahid Beheshti University of Medical Sciences, Tehran, Iran; ^10^ Faculty of Medicine, Mashhad University of Medical Sciences, Mashhad, Iran; ^11^ Department of Nursing, Sabzevar Branch, Islamic Azad University, Sabzevar, Iran; ^12^ College of medicine, University of Arizona, Tucson, AZ, United States; ^13^ School of Medicine, Shahid Beheshti University of Medical Sciences, Tehran, Iran

**Keywords:** cellular senescence, aging, antioxidants, natural antioxidants, food-derived antioxidants

## Abstract

**Background:**

Cellular senescence, characterized by irreversible cell cycle arrest, plays a pivotal role in ageing and the development of age-related pathologies. Mitigating oxidative stress, a primary contributor to cellular ageing, is crucial for inhibiting the senescence-associated secretory phenotype (SASP). A comparative analysis of synthetic and natural antioxidants is necessary to evaluate the efficacy of synthetic and natural antioxidants in this context.

**Method:**

A systematic review encompassed studies published up to July 2023, utilizing prominent databases such as PubMed, Google Scholar, Scopus, and Web of Science. To enhance the efficiency of data screening and selection, we employed Rayyan. ai, an advanced tool designed for systematic reviews.

**Result:**

The review encompassed 33 studies examining the impact of diverse antioxidants on cellular senescence. Findings indicated that synthetic antioxidants, such as N-acetylcysteine, and natural alternatives, like Vitamin C, demonstrated efficacy in attenuating oxidative stress and senescence markers. Notably, natural antioxidants frequently exhibited comparable or superior efficacy to their synthetic counterparts in most studies. Furthermore, the synergistic effects of antioxidant combinations sometimes yield enhanced benefits. It is worth noting that certain recently developed synthetic compounds, such as MHY2233, have shown promising results, exhibiting greater potency than established antioxidants in mitigating senescence markers.

**Conclusion:**

Dietary practices and the aging process can influence these intricate processes and how they interact, serving as potential primary and secondary preventative strategies. The Mediterranean diet, dietary antioxidants, and limiting calorie intake are promising nutritional strategies. Better insight into the molecular mechanisms of aging may facilitate the development of efficient biomarkers and antioxidants for diagnosis or treatment.

**Systematic Review Registration:**

https://osf.io/b67wm/

## 1 Introduction

Cellular ageing or senescence, a process that induces irreversible cell cycle arrest, is a cellular response to various stresses (Qian and Chen, 2010). It is known as an important factor in tumor suppression and ageing (Parrinello et al., 2003), and age-related diseases such as osteoarthritis (Martin et al., 2004), glaucoma (Liton et al., 2005) the diabetic pancreas (Shimizu et al., 2012), and neurodegenerative disorders (Chen, 2000). Thus, it is a possible therapeutic target for age-related conditions (Childs et al., 2015). Cellular senescence can be classified depending on its inducing factors into replicative senescence (RS), stress-induced premature senescence (SIPS), drug-induced senescence, and oncogene-induced ageing (Dierick et al., 2002).

When cells enter the senescent stage, growth is arrested, cells undergo dramatic morphological changes, and they express senescence markers such as senescence-associated β-galactosidase *(SA-β-gal)*, *p16INK4, p53*, and *p21*, which leads to the formation of DNA segments with chromatin alterations reinforcing senescence (DNA-SCARS) (Campisi, 2013; Giordano and Terlecky, 2012). Recently, it has been demonstrated that senescent cells express the senescence-associated secretory phenotype (SASP), which is released into the environment and impacts the functions of neighboring cells (Coppe et al., 2010). The SASP includes various transcription factors, growth factors, proinflammatory cytokines, including *Interleukin (IL)-1, IL-6*, and *IL-8*, proteases, and matrix metalloproteinases (Rodier and Campisi, 2011). Since many of these molecules are proinflammatory, SASP production could potentially provoke inflammation and play a significant role in numerous age-related diseases such as Alzheimer’s (Bagyinszky et al., 2017). Ageing cells accumulate DNA damage, which may result in irreversible growth arrest (Lim, 2006). Cell cycle checkpoints sense damage to the DNA structure and trigger complex cellular repair responses. If the DNA cannot be repaired effectively, cells go into permanent cell cycle arrest and apoptosis. A permanent cell cycle arrest occurs with cells remaining in the G0/G1 phase during senescence (Bartek and Lukas, 2001).

It has been reported that many signalling pathways, such as the phosphatidylinositol 3-kinase (PI3K)/AKT/mammalian target of rapamycin (*mTOR), p53-p21*, and *p16-pRb*, mainly regulate cellular ageing (Liu and Sabatini, 2020; Legrain et al., 2014). Several causes are important in cell aging, including glycation, mutation, protein aggregation, telomere shortening, and oxidative stress (Hayflick, 2007; Liochev, 2015). It has been suggested that telomere shortening is an important mechanism in cellular ageing (Passos and von Zglinicki, 2005). DNA damage is associated with the modulation of mammalian Sirtuin (SIRT), nicotinamide adenosine dinucleotide (NAD)-dependent protein deacetylase, which regulates multiple cellular processes, including senescence, metabolism, and cell cycle (Ma et al., 2019; Watroba et al., 2017). SIRT modulates cellular senescence through the deacetylation of various signalling molecules, such as Nuclear Factor-κB (NF-κB), forkhead box O (FOXO), and *p53* (Inoue et al., 2007).

Oxidative stress is an important cause of cellular ageing and may lead to pathological damage to cells and cell death (Hayflick, 2007; Liochev, 2015). Oxidative stress can cause DNA damage, including DNA double-strand breaks, promote phosphorylation of *γ-H2AX*, activate ATM kinase activity, upregulate *p53/p21* activity, and initiate cellular ageing (Rai et al., 2009). According to Denhan Harman’s oxidative stress theory of ageing, cells undergo ageing due to oxidative stress caused by free radicals (Harman, 1956). When the production of free radicals and reactive oxygen species (ROS) exceeds the capacity of the cells' antioxidant mechanisms, oxidative stress usually occurs (Liu et al., 2014). This phenomenon leads to oxidative damage to macromolecules like proteins, lipids, and DNA, contributing to cellular dysfunction and the progression of cell ageing (Colavitti and Finkel, 2005).

Antioxidants are compounds that interact with reactive oxygen species and their derivatives to prevent them from harming the cells. They are classified according to their sources into two main groups: synthetic and natural antioxidants (Amarachukwu Uzombah, 2022). Synthetic antioxidants are synthesized artificially by combinations of chemical substances. They are chemically synthesized compounds since they do not occur in nature and are added to food as preservatives to help prevent lipid oxidation (Atta et al., 2017). Natural antioxidants, explicitly derived from food and medicinal plants, including fruits, vegetables, spices, cereals, and traditional botanicals, may be considered bioactive compounds (Xu et al., 2017; Deng et al., 2012). Natural antioxidants in foods seem to provide metabolic benefits and are associated with a lower risk of developing several health problems (Comert and Gokmen, 2018). Furthermore, the protective effects of antioxidants in fruits and vegetables are related to three main groups: carotenoids, phenolic compounds, and vitamins (Thaipong et al., 2006).

The defense against oxidants declines during the ageing process (Muthuswamy et al., 2006), and it can be enhanced by supplementing antioxidants (Kumaran et al., 2009). Antioxidants consistently inhibit the induction of senescence phenotypes and increasing antioxidant defenses delays ageing by lowering oxidative stress (Nestelbacher et al., 2000; Agarwal et al., 2005). They are important to prevent cellular ageing (Syslova et al., 2014). Antioxidant production may be an effective, preventive, and therapeutic method for the ageing process (He et al., 2019; Zamora-Ros et al., 2010). Few systematic reviews have conducted precise comparisons between synthetic and natural antioxidants. This systematic review aims to critically compare the efficacy, mechanisms, and translational relevance of synthetic versus natural antioxidants in mitigating cellular senescence, with the goal of identifying promising candidates and therapeutic strategies for age-related interventions.

## 2 Methods

The current study is a systematic review and meta-analysis that adheres to the principles outlined in the PRISMA checklist (Moher et al., 2009). The study protocol has been registered within the Open Science Framework (OSF) (DOI: 10.17605/OSF.IO/B67WM).

### 2.1 Search strategy

In this systematic review, we conducted a comprehensive literature search across four significant databases: PubMed, Google Scholar, Scopus, and Web of Science (ISI). Up to July 2023, we identified 6,507 original articles. The search strategy and keywords used in each database are detailed in Table 1.

**TABLE 1:** Search strategies and result of the searching procedure.

Data base	Search strategy
PubMed	“Synthetic antioxidants” [tiab] OR “Natural antioxidants” [tiab] OR “antioxidants” [tiab] OR “BHA” [tiab] OR “butylated hydroxyanisole” [tiab] OR “BHT” [tiab] OR “butylated hydroxytoluene” [tiab] OR “PG” [tiab] OR “Propyl Gallate” [tiab] OR “OG” [tiab] OR “Octyl Gallate” [tiab] OR “DG” [tiab] OR “Dodecyl Gallate” [tiab] OR “EDTA” [tiab] OR “Ethylenediaminetetraacetat” [tiab] OR “Ethylenediaminetetraacetic acid” [tiab] OR “TBHQ” [tiab] OR “tertiary butylhydroquinone” [tiab] OR “Acetylcysteine” [tiab] OR “Tocopherols” [tiab] OR “Ascorbic Acid” [tiab] OR “Rosemary extract” [tiab] OR “Anthocyanins” [tiab] OR “Lycopene” [tiab] OR “Carotenoids” [tiab] OR “Flavonoid” [tiab] OR “Vitamin C” [tiab] OR “Vitamin E” [tiab] OR “Tocopherol” [tiab] OR “tocotrienols” [tiab] OR “Vitamin A” [tiab] OR “Carotenoids” [tiab] OR “Carotene” [tiab] OR “Zeaxanthin” [tiab] OR “Lutein” [tiab] OR “Lycopene” [tiab] OR “beta-Cryptoxanthin” [tiab] OR “beta cryptoxanthin” [tiab] OR “Polyphenols” [tiab] OR “flavonols” [tiab] OR “flavanols” [tiab] OR “Catechins” [tiab] OR “actocyanins” [tiab] OR “isoflavones” [tiab] OR “Phenolic acids” [tiab] OR 2 “Phenolics” [tiab] OR “trace elements” [tiab] OR “selenium” [tiab] OR “Zinc” [tiab] OR “Ellagic acid” [tiab] OR “gallic acid” [tiab] OR “3,4,5-trihydroxybenzoic acid” [tiab] OR “protocatechuic” [tiab] OR “p-hydroxybenzoic acids” [tiab] OR “melanoidins” [tiab] OR “anthocyanins” [tiab] OR “Thymoquinone” [tiab] OR “Carvacrol” [tiab] OR “t-anethole” [tiab] OR “4-terpineol” [tiab] OR “Sesamin sesamolin” [tiab] OR “Curcumin” [tiab] OR “Monoterpenoid” [tiab] OR “Linalool” [tiab] OR “beta-carotene” [tiab] OR “beta carotene” [tiab] 686831 results 2- “Cellular aging” [tiab] OR “Erythrocyte Aging” [tiab] OR “Senescence-Associated secretory phenotype” [tiab] OR “Telomere shortening” [tiab] OR “cellular senescence” [Me SH] 30406 results ≠ 1 AND ≠ 2
WOS	((TS=(“synthetic antioxidant”) OR TS=(“synthetic antioxidants”) OR TS=(BHA) OR TS=(“butylated hydroxyanisole”) OR TS=(BHT) OR TS=(“butylated hydroxytoluene”) OR TS=(PG) OR TS=(“Propyl Gallate”) OR TS=(OG) OR TS=(“Octyl Gallate”) OR TS=(DG) OR TS=(“Dodecyl Gallate”) OR TS=(EDTA) OR TS=(“Ethylenediamine tetra acetate”) OR TS=(“Ethylenediaminetetraacetic acid”) OR TS=(TBHQ) OR TS=(“tertiary butylhydroquinone”) OR TS=(Acetylcysteine)) OR (TS=(“natural food derived antioxidants”) OR TS=(“natural food-derived antioxidants”) OR TS=(“natural food derived antioxidant”) OR TS=(“natural food-derived antioxidant”) OR TS=(“natural antioxidants”) OR TS=(“natural antioxidant”) OR TS=(Tocopherol*) OR TS=(“Ascorbic Acid”) OR TS=(“Rosemary Extract”) OR TS=(Anthocyanin*) OR TS=(lycopene) OR TS=(Carotenoid*) OR TS=(Flavonoid*) OR TS=(“Vitamin C″) OR TS=(“ascorbic acid”) OR TS=(ascorbate) OR TS=(“Vitamin E”) OR TS=(tocopherol*) OR TS=(tocotrienol*) OR TS=(“Vitamin A″) OR TS=(Carotenoid*) OR TS=(carotene*) OR TS=(zeaxanthin) OR TS=(lutein) OR TS=(lycopene) OR TS=(β-cryptoxanthin) OR TS=(“beta cryptoxanthin”) OR TS=(Polyphenol*) OR TS=(flavonol*) OR TS=(flavanol*) OR TS=(catechin*) OR TS=(anthocyanin*) OR TS=(isoflavone*) OR TS=(“phenolic acid”) OR TS=(“phenolic acids”) OR TS=(Phenolic*) OR TS=(“Trace elements”) OR TS=(selenium) OR TS=(zinc) OR TS=(“Ellagic acid”) OR TS=(“gallic acid”) OR TS=(“3,4,5-trihydroxybenzoic acid”) OR TS=(protocatechuic) OR TS=(“p-hydroxybenzoic acid”) OR TS=(“p-hydroxybenzoic acids”) OR TS=(melanoidin*) OR TS=(Anthocyanin*) OR TS=(Thymoquinone) OR TS=(carvacrol) OR TS=(t-anethole) OR TS=(4-terpineol) OR TS=(“Sesamin sesamolin”) OR TS=(Curcumin) OR TS=(Monoterpenoid*) OR TS=(Linalool) OR TS=(β-carotene*) OR TS=(“beta carotene”) OR TS=(“beta carotenes”))) AND (TS=(“Cellular Aging”) OR TS=(“Cellular Senescence”) OR TS=(“Cell Senescence”) OR TS=(“Cell Aging”) OR TS=(“Cellular Ageing”) OR TS=(“Cellular Aging”) OR TS=(“Erythrocyte Aging”) OR TS=(“Erythrocyte Ageing”) OR TS=(“Telomere Shortening”) OR TS=(“Senescence-Associated Secretory Phenotype”))
Scopus	(((TITLE-ABS-KEY (“synthetic antioxidant”) OR TITLE-ABS-KEY (“synthetic antioxidants”) OR TITLE-ABS-KEY (BHA) OR TITLE-ABS-KEY (“butylated hydroxyanisole”) OR TITLE-ABS-KEY (BHT) OR TITLE-ABS-KEY (“butylated hydroxytoluene”) OR TITLE-ABS-KEY (PG) OR TITLE-ABS-KEY (“Propyl Gallate”) ( OR TITLE-ABS-KEY (OG) OR TITLE-ABS-KEY (“Octyl Gallate”) OR TITLE-ABS-KEY (DG) OR TITLE-ABS-KEY (“Dodecyl Gallate”) OR TITLE-ABS-KEY (EDTA) OR TITLE-ABS-KEY (“Ethylenediamine tetra acetate”) OR TITLE-ABS-KEY (“Ethylenediaminetetraacetic acid”) OR TITLE-ABS-KEY (TBHQ) OR TITLE-ABS-KEY ( “tertiary butylhydroquinone”) OR TITLE-ABS-KEY (Acetylcysteine)) OR (TITLE-ABS-KEY (“natural food derived antioxidants”) OR TITLE-ABS-KEY (“natural food derived antioxidant”) OR TITLE-ABS-KEY ( “natural antioxidant”) OR TITLE-ABS-KEY (“natural antioxidants”) OR TITLE-ABS-KEY (Tocopherol*) OR TITLE-ABS-KEY (“Ascorbic Acid”) OR TITLE-ABS-KEY (“Rosemary Extract”) OR TITLE-ABS-KEY ( Anthocyanin*) OR TITLE-ABS-KEY (lycopene) OR TITLE-ABS-KEY (Carotenoid*) OR TITLE-ABS-KEY ( Flavonoid*) OR TITLE-ABS-KEY (“Vitamin C”) OR TITLE-ABS-KEY (“ascorbic acid”) OR TITLE-ABS-KEY (ascorbate) OR TITLE-ABS-KEY (“Vitamin E”) OR TITLE-ABS-KEY (tocopherol*) OR TITLE-ABS-KEY ( tocotrienol*) OR TITLE-ABS-KEY (“Vitamin A”) OR TITLE-ABS-KEY (Carotenoid*) OR TITLE-ABS-KEY ( carotene*) OR TITLE-ABS-KEY (zeaxanthin) OR TITLE-ABS-KEY (lutein) OR TITLE-ABS-KEY (lycopene) OR TITLE-ABS-KEY (“β-cryptoxanthin”) OR TITLE-ABS-KEY (“beta cryptoxanthin”) OR TITLE-ABS-KEY ( Polyphenol*) OR TITLE-ABS-KEY (flavonol*) OR TITLE-ABS-KEY (flavanol*) OR TITLE-ABS-KEY (catechin*) OR TITLE-ABS-KEY (anthocyanin*) OR TITLE-ABS-KEY (isoflavone*) OR TITLE-ABS-KEY ( “phenolic acid”) OR TITLE-ABS-KEY (“phenolic acids”) OR TITLE-ABS-KEY (Phenolic*) OR TITLE-ABS-KEY (“Trace elements”) OR TITLE-ABS-KEY (selenium) OR TITLE-ABS-KEY (zinc) OR TITLE-ABS-KEY (“Ellagic acid”) OR TITLE-ABS-KEY (“gallic acid”) OR TITLE-ABS-KEY (“3,4,5-trihydroxybenzoic acid”) OR TITLE-ABS-KEY (protocatechuic) OR TITLE-ABS-KEY (“p-hydroxybenzoic acid”) OR TITLE-ABS-KEY (“p-hydroxybenzoic acids”) OR TITLE-ABS-KEY (melanoidin*) OR TITLE-ABS-KEY (Anthocyanin*) OR TITLE-ABS-KEY (Thymoquinone) OR TITLE-ABS-KEY (carvacrol) OR TITLE-ABS-KEY (“t-anethole”) OR TITLE-ABS-KEY (“4-terpineol”) OR TITLE-ABS-KEY (“Sesamin sesamolin”) OR TITLE-ABS-KEY (Curcumin) OR TITLE-ABS-KEY (Monoterpenoid*) OR TITLE-ABS-KEY (Linalool) OR TITLE-ABS-KEY (“β-carotene”) OR TITLE-ABS-KEY (“β-carotenes”) OR TITLE-ABS-KEY (“beta carotene”) OR TITLE-ABS-KEY (“beta carotenes”))) AND (TITLE-ABS-KEY (“Cellular Aging”) OR TITLE-ABS-KEY (“Cellular Senescence”) OR TITLE-ABS-KEY (“Cell Senescence”) OR TITLE-ABS-KEY (“Cell Aging”) OR TITLE-ABS-KEY (“Cellular Ageing”) OR TITLE-ABS-KEY (“Cellular Aging”) OR TITLE-ABS-KEY (“Erythrocyte Aging”) OR TITLE-ABS-KEY (“Erythrocyte Ageing”) OR TITLE-ABS-KEY (“Telomere Shortening”) OR TITLE-ABS-KEY ( “Senescence-Associated Secretory Phenotype”)))

### 2.2 Eligibility criteria

For this systematic review, we included original research articles published in English that reported on *in vitro* and *in vivo* studies. The focus was on studies examining the effects of both natural and synthetic antioxidants on cellular senescence.

### 2.3 Data extraction

We utilized Rayyan. ai, a web-based tool designed to facilitate the screening process in systematic reviews through semi-automation and collaboration. Rayyan. ai allows researchers to efficiently manage and categorize citations by providing features such as duplicate detection and collaborative labelling (Ouzzani et al., 2016).

Two reviewers (FE and SEM) independently screened the studies, and a third reviewer (MAA) resolved any conflicts. We extracted data according to predefined variables and compiled the information in Supplementary Table S1.

Table 2 summarizes key information from the included studies, encompassing Author(s) and publication year, Country of origin, Study type (*in vitro* or *in vivo*), Antioxidant types and concentrations, Species and cell types investigated, Mechanisms of action, Outcomes related to cellular ageing, and Main conclusions.

**TABLE 2:** Classification of Antioxidants Used Across Studies; In the reviewed studies, antioxidants were categorized based on their chemical properties and natural origin. These categories include synthetic compounds, vitamins, polyphenols, amino acid derivatives, plant extracts, mushroom-derived substances, and other bioactive agents. For each group, we have highlighted representative examples, outlined their typical mechanisms of action, and summarized the primary molecular pathways or biological effects they influence.

References	Class	Examples	Mechanism of action	Target pathways/Effects
(Lamichane et al., 2019), (Khan et al., 2017; Karthikeyan et al., 2024; Hulmi et al., 2016))	Synthetic Antioxidants	NAC, Metformin, Rapamycin, TEMPOL, Setanaxib, MHY2233	ROS scavenging, mTOR inhibition, mitochondrial protection	mTOR pathway, Nrf2 activation, oxidative stress reduction
(Li et al., 2016), (Ren et al., 2022), (Shi et al., 2024), (Khor et al., 2017))	Vitamins	Vitamin C, Vitamin E (α-tocopherol), α-lipoic acid, tocotrienol-rich fraction, nicotinamide mononucleotide (NMN)	Antioxidant activity, NAD+ boosting, mitochondrial support	Oxidative stress, sirtuin pathway, metabolic enhancement
(Shiwakoti et al., 2022), (Coughlan et al., 2016)	Polyphenols	Resveratrol, (−)-epigallocatechin gallate (EGCG), Curcumin, TSG	Free radical scavenging, epigenetic modulation, anti-inflammatory action	NF-κB, AMPK activation, inflammation, apoptosis regulation
Chandra et al. (2022)	Amino Acids &amp; Derivatives	Spermidine, Acetyl-L-carnitine, L-arginine	Autophagy induction, mitochondrial function, NO production	Autophagy, mitochondrial biogenesis, vasodilation
Zhang et al. (2021)	Plant Extracts	Ginsenoside Rg1, Mulberry leaf extract, Ethanolic extract of okra fruit	Anti-inflammatory, insulin sensitization, antioxidant	PI3K/Akt, Nrf2, glucose metabolism, inflammation
Zhong et al. (2024)	Mushroom-Derived Compounds	Clitocybin A, B, and C	Neuroprotective, antioxidant, anti-aging	Nrf2, MAPK, mitochondrial stabilization
(Wang et al., 2019), (Zhang et al., 2024)	Other Bioactive Compounds	Caffeine, Cordycepin, Rotenone, 17β-estradiol	CNS stimulation, AMPK activation, estrogen receptor signaling	Adenosine receptor antagonism, apoptosis, hormonal regulation

### 2.4 Quality assessment

Two independent reviewers assessed the quality of primary studies using the JBI Critical Appraisal tool. Any disagreement between the two reviewers was solved by mutual consensus, and then a third reviewer independently scored it.

## 3 Results

### 3.1 Study selection

**FIGURE 1:**
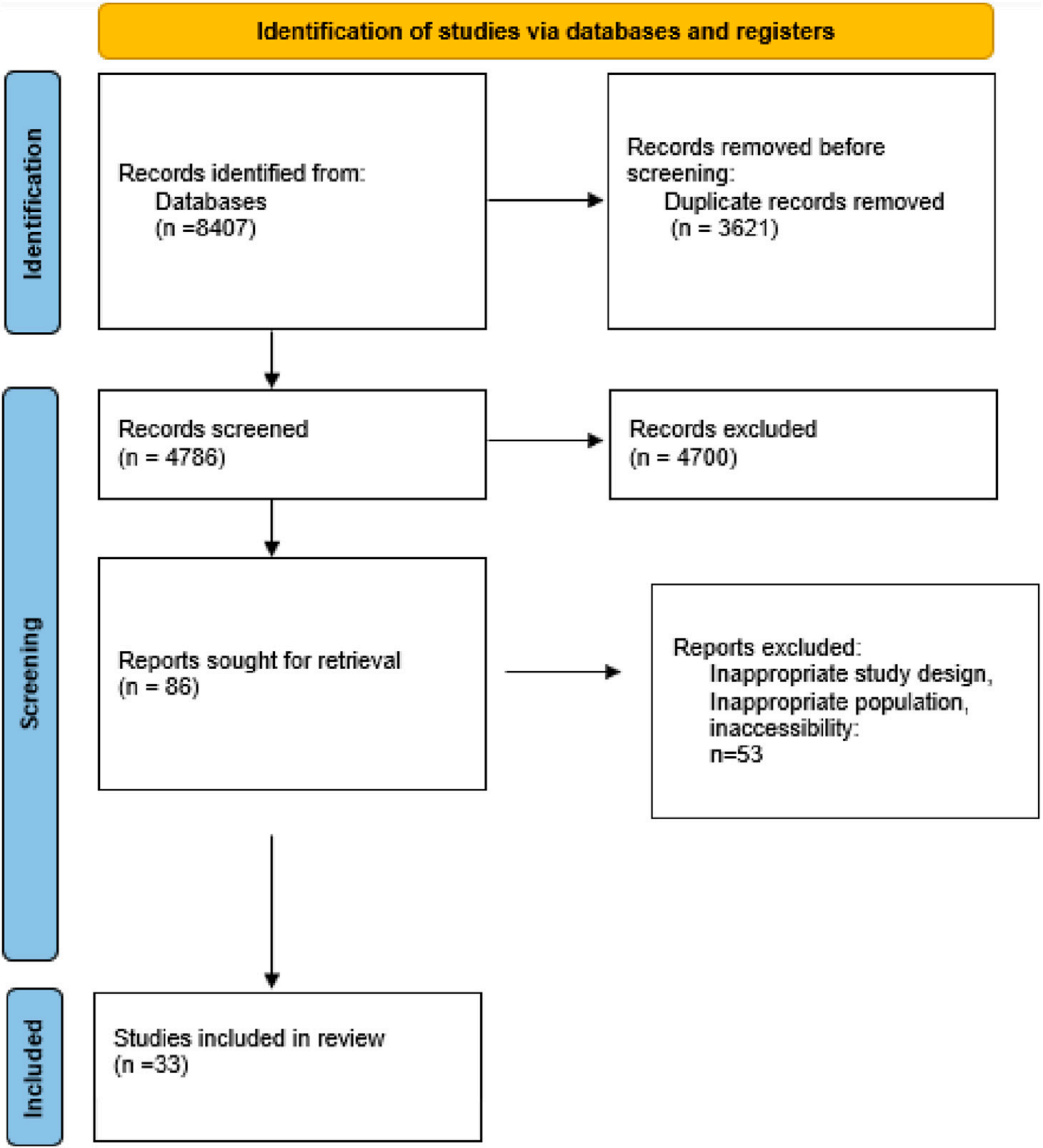
PRISMA flow chart of study selection procedure.

Our initial search across the selected databases yielded 6,507 articles. After removing some duplicates and conducting a preliminary screening of titles and abstracts, we identified 119 articles as relevant for further evaluation. Following a detailed assessment, 44 duplicate articles were excluded, resulting in a final set of 75 studies for detailed analysis. Among these 75 studies, only 33 articles met our criteria by examining natural and synthetic antioxidants' effects on cellular senescence. Consequently, 42 articles were excluded for focusing solely on one type of antioxidant or not addressing cellular senescence adequately. These 33 studies were retained for detailed analysis and inclusion in our systematic review. This rigorous selection process ensured that our review focused on studies that comprehensively addressed the comparative effects of both antioxidants on cellular ageing processes (Figure 1).

### 3.2 Study characteristics

This systematic review included 33 studies exploring various antioxidants' effects on cellular senescence and ageing (Supplementary Table S1) (Bhayadia et al., 2016; Bjelica et al., 2019; Briganti et al., 2008; Che et al., 2022; Chen et al., 2023; Chen et al., 2020; Dai et al., 2022; Jumnongprakhon et al., 2021; Khan et al., 2017; Khatri et al., 2016; Lamichane et al., 2019; Lee and Bae, 2014; Lee et al., 2013; Li et al., 2021; Li et al., 2016; Moon et al., 2009; Okuni et al., 2022; Pan et al., 2022; Ren et al., 2022; Senthil et al., 2016; Shen et al., 2020; Shiwakoti et al., 2022; Wang et al., 2022; Wang et al., 2018; Xu T. et al., 2019; Xu TZ. et al., 2019; Yang et al., 2018; Yu et al., 2011; Zhou et al., 2020; Zhu et al., 2023; Sekelova et al., 2023; Coughlan et al., 2016; Zhang et al., 2024). Most of these studies were conducted in Asian countries, particularly China, with some research also taking place in European nations such as Austria, Italy, Germany, and Serbia. One study was a collaborative effort between Taiwan and the USA. The studies were published between 2008 and 2023, showcasing a range of recent research in this field. The research methodologies employed in these studies varied. Of the 33 studies, 15 were conducted exclusively *in vitro*, 12 combined both *in vitro* and *in vivo* approaches, four were solely *in vivo* experiments, and two utilized both *ex vivo* and *in vitro* methods.

**FIGURE 2:**
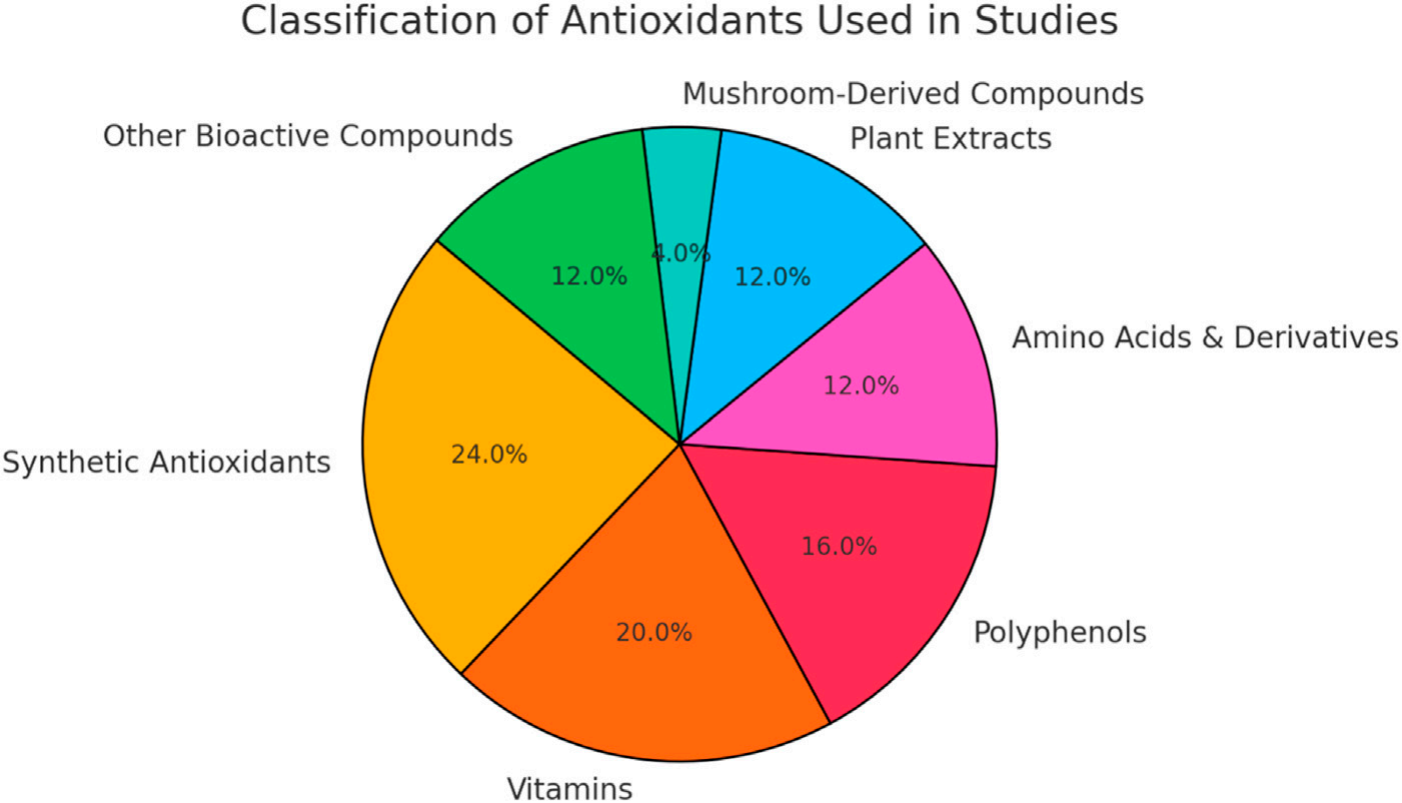
Distribution of antioxidant classes included in literature. Synthetic antioxidants and vitamins, followed by polyphenols were more repeated in the studies included.

These studies investigated a diverse array of antioxidants, encompassing both synthetic and natural compounds. Synthetic antioxidants such as N-acetylcysteine (NAC), Metformin, Rapamycin, 4-hydroxy-2,2,6,6-tetramethylpiperidin-1-oxyl (TEMPOL), Setanaxib, and MHY2233 were examined for their potential to mitigate cellular senescence and ageing processes. NAC, in particular, was a common choice across many studies (Table 2) (Figure 2).

Natural antioxidants featured prominently in the research, including a broad spectrum of compounds such as vitamins (Vitamin C (ascorbic acid), Vitamin E (α-tocopherol) and other vitamin-related compounds including α-lipoic acid, tocotrienol-rich fraction, and nicotinamide mononucleotide), polyphenols (Resveratrol (−)-epigallocatechin gallate (EGCG), and curcumin), plant extracts (ginsenoside Rg1, mulberry leaf extract, and ethanolic extract of okra fruit), Amino Acids and Derivatives (spermidine, acetyl-L-carnitine, and L-arginine), mushroom-derived Compounds (Clitocybins A, B, and C from the mushroom Clitocybe aurantiaca), and other Bioactive Compounds (caffeine, cordycepin, ancient M, rotenone, and 17β-estradiol (E2)). Often researched for their intricate compositions, these natural substances might simultaneously target several pathways, therefore improving their effectiveness in anti-ageing efforts.

The research looked at how these antioxidants affected different species and cell types. Among the most often used human cells were fibroblasts, endothelial cells, stem cells, and cancer cell lines. Especially *in vivo* research, animal models—especially mice and rats—were often used. Some studies even included other species including *Caenorhabditis elegans* nematodes.

Specific cell types, such as human dermal fibroblasts, umbilical vein endothelial cells, mesenchymal stem cells, and various organ-specific cells, such as chondrocytes, neurons, and hepatocytes, were studied. Animal studies looked at tissues from several organs including the brain, liver, and kidney.

Reflecting the different character of the chemicals and experimental designs, studies showed great variation in the concentrations and dosages of antioxidants. NAC levels, for example, usually varied from 1 mM to 10 mM *in vitro*; *in vivo* dosages were usually about 100–150 mg/kg/day. Several studies looked at natural and synthetic micromolar range chemicals (Suzuki et al., 1991; Chen et al., 2008; Park and Kim, 2012).

### 3.3 Mechanisms

Though much has been learned about cellular senescence, senescent cell *in vivo* detection and assessment still present difficulties. The markers that show *in vitro* efficacy, such as the lack of DNA synthesis, are less reliable in living organisms because of the prevalence of quiescent or terminally differentiated cells (Dierick et al., 2002; Lim, 2006; Liochev, 2015; Comert and Gokmen, 2018; Dai et al., 2022). Furthermore, the morphological alterations seen in cell cultures frequently do not appear *in vivo*, and cell cycle markers can be deceptive because of inflammation or particular cell types. Accurate evaluation is made even more difficult by the low number of senescent cells and the natural complexity of living systems. Research papers, on the other hand, showed actively employing more strong techniques for identifying senescence in both live animals and fixed tissues (Sekelova et al., 2023).

Reducing Oxidative Stress Nearly all studies found lower oxidative stress, mostly by lowering ROS levels. Various synthetic antioxidants—such as NAC, Tempol—and natural ones—such as Apocynin (APO), Vitamin C, Resveratrol—helped to accomplish this. Many studies also found higher expression or activity of antioxidant enzymes such as superoxide dismutase (SOD), catalase, and glutathione peroxidase (GPx).

Often assessed by SA-\u03b2-gal activity, many studies found lower lysosomal compartment and activity. This is a well-known sign of cellular senescence.

Many studies found that the p16-pRB and p53-p21 axes, vital pathways in cell cycle arrest and senescence, were deactivated. Other studies found cell proliferation to be preserved, usually assessed by Ki-67 protein expression or ethynyl-2′-deoxyuridine (EdU) incorporation.

Many studies found less DNA damage, usually assessed by lower γ-H2AX foci or 8-Hydroxy-2′-deoxyguanosine (8-OHdG) levels (Senthil et al., 2016; Shen et al., 2020; Wang et al., 2022; Wang et al., 2018; Xu T. et al., 2019; Xu TZ. et al., 2019; Yang et al., 2018; Wilcox, 2010; Thannickal et al., 2023; Sultana et al., 2024).

Many studies found lower cytokine release, especially for pro-inflammatory ones like IL-1β, IL-6, IL-8, and Tumor necrosis factor (TNF)-α. This handles the SASP.

Some studies found better mitochondrial function, such higher mitochondrial membrane potential and Adenosine triphosphate (ATP) levels (Legrain et al., 2014; Inoue et al., 2007; Kumaran et al., 2009; He et al., 2019; Jumnongprakhon et al., 2021).

Extracellular Matrix Maintenance: Maintenance of extracellular matrix components (e.g., collagen II, aggrecan) and inhibition of matrix-degrading enzymes (e.g., matrix metalloproteinases (MMPs), A Disintegrin And Metalloproteinase with Thrombospondin motifs (ADAMTS)) was reported in several studies, especially those concentrating on *in vivo* experiments and cartilage or intervertebral discs.

Some studies found that various signalling pathways—including NF-\u03baB, AMP-activated protein kinase (AMPK), SIRT1, nuclear factor erythroid two related factor 2 (Nrf2) - Kelch-like ECH-associated protein 1 (Keap1), and mitogen-activated protein kinase (MAPK)—were modulated.

Some studies found telomere maintenance effects, such as higher telomerase activity and telomerase reverse transcriptase (TERT) expression. Some studies found autophagy modulation, usually by means of AMPK or Bcl-2-Beclin1 interaction controls. Many studies found a decrease in apoptosis, usually together with preservation of cell viability.

Stem cell studies revealed maintenance of stem cell markers—e.g., octamer-binding transcription factor 4 (OCT4), NANOG, SRY (sex determining region Y)-box 2 (SOX2).

Some studies—especially those using Ginsenoside Rg1 or APO—showed suppression of the Nucleotide-binding oligomerization domain, leucine-rich Repeat, and Pyrin domain containing 1 (NLRP1) or NLRP3 inflammasome activation (Moon et al., 2009; Mursaleen et al., 2020; Marashi et al., 2024).

### 3.4 Effects on cellular ageing

Consistently lowering ROS levels, nearly all synthetic antioxidants—e.g., NAC, TEMPOL—and natural—e.g., Vitamin C, Resveratrol, Ginsenoside Rg1—reduced oxidative stress. Often, this was coupled with lower lipid peroxidation and higher antioxidant enzyme activity (SOD, catalase, glutathione peroxidase).

Most antioxidants lowered SA-\u03b2-gal activity, a major indicator of cellular senescence. They also lowered the levels of other senescence markers including p16, p21, and p53.

Many antioxidants—including NAC, APO, Plumericin, Vitamin C, Resveratrol—helped preserve cell proliferation and avoided cell cycle arrest in different cell types under senescence-inducing conditions. Often, this went hand in hand with higher cyclin and CDK expression (Liton et al., 2005; Lim, 2006; Liu and Sabatini, 2020; Legrain et al., 2014; Liochev, 2015; Ma et al., 2019; Liu et al., 2014; Kumaran et al., 2009; Jumnongprakhon et al., 2021).

Several chemicals—including NAC, Cordycepin, and Vitamin C—lowered 8-OHdG levels and gamma-H2AX foci.

By lowering the release of pro-inflammatory cytokriines (IL-1\u03b2, IL-6, TNF-\u03b1), many antioxidants, especially natural chemicals like Ginsenoside Rg1 and Cordycepin, suppressed the SASP.

Compared to conventional antioxidants like NAC, some substances, such as nicotinamide mononucleotide (NMN) and 2,3,5,4′-Tetrahydroxystilbene-2-O-\u03b2-D-glucoside (TSG), exhibited better results in maintaining mitochondrial function.

A few substances, including TSG and vitamin C, were said to reduce telomere loss or boost telomerase activity (Dierick et al., 2002; Giordano and Terlecky, 2012; Deng et al., 2012; Dai et al., 2022).

Different antioxidants changed important senescence-related signaling pathways. Many, for instance, activated the AMPK-SIRT1-peroxisome proliferator-activated receptor-gamma coactivator-1α (PGC-1α) and Nrf2-Keap1 pathways while suppressing NF-\u03baB activation.

Some studies found tissue-specific advantages including preservation of bone mineral density (NAC and E2), enhanced endothelial function (TEMPOL and APO combination, caffeine, TSG), or defense against radiation-induced damage (Cordycepin).

Natural chemicals sometimes had equal or better effects to synthetic antioxidants. Ginsenoside Rg1, for example, often outperformed TEMPOL and APO in lowering inflammatory responses and senescence markers.

Several studies found that mixing several antioxidants produced stronger effects. For instance, combining TEMPOL and APO worked more effectively to enhance endothelial function than either compound by itself.

Among the newly created chemicals, MHY2233 was more powerful than Resveratrol in SIRT1 activation and senescence marker reduction (Bagyinszky et al., 2017; Bartek and Lukas, 2001; Amarachukwu Uzombah, 2022; Atta et al., 2017; Bhayadia et al., 2016; Bjelica et al., 2019).

Although most antioxidants had positive effects in lowering cellular senescence, their effectiveness differed with the particular chemical, concentration, cell type, and experimental settings. Natural substances sometimes outperformed or matched synthetic antioxidants, and mixes of several antioxidants occasionally produced greater advantages. While they draw attention to the intricacy of cellular senescence and the necessity of individualized strategies, these results also show the promise of antioxidants in anti-ageing initiatives.

## 4 Discussion

While synthetic and natural antioxidants share common pathways in alleviating oxidative stress and influencing cellular ageing processes, natural antioxidants often confer additional benefits due to their complex composition and ability to target multiple pathways simultaneously. This multifaceted approach potentially renders natural antioxidants more effective in comprehensive anti-ageing strategies. Nonetheless, both categories of antioxidants contribute significantly to our understanding of cellular senescence and offer promising avenues for further cellular ageing research.

### 4.1 Synthetic antioxidants

NAC, as a mucolytic agent, is also generally used in the treatment of acetaminophen overdose and shows good tolerance. However, gastrointestinal complications, including nausea, vomiting, and diarrhea, might happen due to prolonged or high-dose administration. The compound’s distinctive sulfurous smell, like rotten eggs, can worsen these symptoms when taken orally. To address this problem, effervescent-flavored tablets have been provided as an alternative formulation (Mursaleen et al., 2020). The comparative analysis of NAC and different natural antioxidants demonstrates a complex effectiveness in inhibiting cellular senescence and oxidative stress. Comparative NAC and APO analyses have been well-examined in the literature to show their substantial anti-ageing properties in various microenvironments. According to Bjelica et al. (2019), both compounds alleviated hydroxyurea-induced senescence in peripheral blood mesenchymal stromal cells, and APO was slightly more efficient (Bjelica et al., 2019). Lee et al. (2013), Lee et al. (2014) found similar outcomes in cancer cells and fibroblasts, reporting that both antioxidants reduced ROS generation and senescence indicators, with APO specifically inhibiting nicotinamide adenine dinucleotide phosphate (NADPH) oxidase (Lee and Bae, 2014; Lee et al., 2013). A study by Shiwakoti et al. (2022) on porcine coronary artery cells observed that NAC and APO, alongside Resveratrol, prevented nanoplastics-induced oxidative stress and senescence, and APO and resveratrol raised SIRT1 expression more than NAC(67). Yu et al. (2011) further confirmed these results in endothelial cells. They noted that NAC, Rotenone, and APO can maintain cellular proliferation and nitric oxide production of Umbilical vein endothelial cells with similar efficacy (Yu et al., 2011).

### 4.2 Comparative efficacy

While NAC exhibits potent antioxidants and anti-aging capabilities, its efficacy often changes compared to natural alternatives. For example, in the comparison between α-lipoic acid, α-tocopherol, and NAC (Briganti et al., 2008), NAC had slightly lower efficacy than α-lipoic acid in mitigating ROS levels and senescence-associated morphological changes (Briganti et al., 2008). Tocotrienol-rich fraction and alpha-tocopherol (Khor et al., 2017) were more efficient in reducing free radical generation than NAC (Coughlan et al., 2016). Similarly, Wang et al. (2022) revealed that TSG, a natural antioxidant, outperformed NAC in most parameters related to endothelial aging such as increasing telomerase activity, improving mitochondrial function and reducing oxidative stress through the PGC-1α pathway activation, particularly at high concentrations (Wang et al., 2022). Khan et al. (2017) showed that plumericin surpassed NAC in decreasing ROS production, particularly at early times. In contrast, NAC had slightly better results in preserving cell proliferation and inhibiting SASP factor expression (Khan et al., 2017).

Khatri et al. (2016) reported that curcumin outperformed NAC in regenerating aged stromal cell function, and significantly improved multiple markers of cellular health both *in vitro* and *in vivo* (Khatri et al., 2016). Yang et al. (2018) showed similar anti-senescence effects between NAC and ascorbic acid in D-galactose-induced aging of bone marrow mesenchymal stem cells (Yang et al., 2018). In addition, Li et al. (2016) provided a more comprehensive comparison. According to their study, considering several antioxidants, including NAC, metformin, rapamycin, vitamin C, vitamin E, epigallocatechin gallate, and resveratrol, vitamin C appeared to be the most potent substance. Vitamin C was most effective at lower concentrations and performed better than NAC and others in various aspects of cellular rejuvenation (Li et al., 2016). Zhou et al. (2020) found that while NAC and E2 effectively decreased oxidative stress and senescence indicators, E2 had marginally more effects, specifically in reducing the SASP factor (Zhou et al., 2020). Despite these comparisons, NAC’s broad-spectrum efficacy is highlighted through its consistent suppression of ROS levels, attenuation of aging markers, and protection against different cellular stressors across studies, emphasizing its potential antioxidant and anti-senescence effects. NAC, in comparison with spermidine (Che et al., 2022) and the ethanolic extract of Okra fruit (Jumnongprakhon et al., 2021), showed comparable or slightly stronger effects in alleviating oxidative stress and senescence markers (Che et al., 2022; Jumnongprakhon et al., 2021). Furthermore, Moon et al. (2009) reported that NAC had higher protective effects against H2O2-induced cell death than Clitocybins A, B, and C from the mushroom Clitocybe aurantiaca (Moon et al., 2009). Additionally, Pan et al. (2022) found high efficacy of NAC in decreasing intracellular ROS levels of serum-starved adipose-derived stem cells. However, Acetyl-L-carnitine showed better results in other aspects of cellular aging, like inhibiting senescence markers (Pan et al., 2022). In another study by Park and Kim (2012), NAC was better than natural antioxidants in neutralizing ROS, demonstrating superior antioxidant capabilities by completely neutralizing advanced glycation end products (AGE)-induced ROS generation, while L-arginine and sepiapterin did not achieve this level of effectiveness (Sekelova et al., 2023).

### 4.3 Safety and tolerability

While metformin, as a common medication for type 2 diabetes management, generally demonstrates safety in long-term usage, it may produce probable side effects in the gastrointestinal system, such as diarrhea and nausea (Ashraf et al., 2022). In a recent comparative analysis by Zhu et al. (2023), the efficacy of the synthetic antioxidant metformin against natural alternatives, specifically Vitamin E and Mulberry leaf extract (MLE), was evaluated. The findings showed that MLE, particularly at higher concentrations, showed comparable effectiveness to metformin in decreasing oxidative stress and cellular senescence, suggesting its potential as a natural substitute (Zhu et al., 2023).

The nitroxide antioxidant TEMPOL is recognized for its radioprotective properties and is generally assumed to be safe at therapeutic doses, although higher concentrations can lead to hypotension and cardiovascular effects. Although the safety of its short-term utilization is somewhat well-established, the long-term consequences of extended TEMPOL are unknown, and further research is warranted.

### 4.4 Mechanistic insights

TEMPOL’s action mechanism consists of redox cycling, mimicking SOD activity and increasing nitric oxide bioavailability. This antioxidant effectively protects mitochondria from oxidative damage, enhances tissue oxygenation, lowers insulin resistance, and reduces ischemia/reperfusion injury in critical tissues such as the heart and brain (Wilcox, 2010). In a comparative study, Chen et al. (2019) investigated the effects of TEMPOL against natural antioxidants Ginsenoside Rg1 and APO. Although all examined antioxidants mitigated ROS levels, Ginsenoside Rg1 at a dose of 10 mg/kg exhibited higher efficacy in reducing oxidative stress, preventing inflammasome markers and ageing-related neuronal damage compared to TEMPOL and APO in neurons of the brain cortex and hippocampus of Senescence-accelerated mouse prone 8 (SAMP8) mice (Chen et al., 2020).

### 4.5 Combination strategies

Interestingly, TEMPOL combined with natural antioxidants can potentially increase overall antioxidant efficacy, thus resulting in synergistic benefits. Research by Bhayadia et al. (2016) showed that combining synthetic and natural antioxidants may yield superior results in specific scenarios. Their study reported that while TEMPOL alone did not enhance vasodilation, its combination with the natural antioxidant APO significantly improved endothelium-dependent vasodilation in aortic rings from aged mice. This synergistic approach might offer higher overall antioxidant efficacy (Bhayadia et al., 2016). These findings underscore the complexity of antioxidant therapy and highlight the need for continued research to optimize the use of both synthetic and natural antioxidants in addressing age-related oxidative stress and its associated pathologies.

Setanaxib, an NADPH oxidase inhibitor, is currently being evaluated for its potential therapeutic effects in multiple conditions. While the detailed long-term safety profile is unclear, pre-clinical data have shown the ability of setanaxib in treating primary biliary cholangitis (PBC) via its anti-fibrotic and anti-inflammatory effects. Setanaxib was evaluated in a phase 2 trial for PBC and showed good tolerance and potential in alleviating liver stiffness and fatigue. However, it did not achieve the primary endpoint of significantly reducing gamma-glutamyl transferase (GGT) levels (Invernizzi et al., 2023). Pre-clinical evidence supports setanaxib’s effectiveness in lowering ROS generation and fibrotic effects in liver, kidney, and lung models. However, more research is necessary to completely clarify these discoveries' therapeutic potential and long-term effects on human health before they can be used in clinical practice (Thannickal et al., 2023). Dai et al. (2022) conducted a comparative study to assess the efficacy of the synthetic antioxidant Setanaxib against the natural antioxidant APO. They found that both compounds blocked the NADPH oxidase 4 (NOX-4) pathway, effectively decreased cell senescence and decelerated intervertebral disc degeneration, and Setanaxib showed comparable effectiveness to APO (Dai et al., 2022).

The safety profile of MHY2233, a relatively new compound, has limited publicly available data. While preclinical studies show possible antioxidant effects, its human safety profile has insufficient data. Therefore, more study is necessary to determine its safety and effectiveness during long-term use (Lamichane et al., 2019). Lamichane et al. (2019) conducted a study and revealed higher efficacy of MHY2233 than Resveratrol in increasing SIRT1 deacetylase activity, reducing markers of cellular ageing, and protecting against oxidative stress-induced damage. These promising outcomes make MHY2233 as a candidate for addressing vascular ageing and related pathologies. Furthermore, their study demonstrated the stronger property of MHY2233 in preventing senescence of endothelial progenitor cells compared to the natural antioxidant Resveratrol (Lamichane et al., 2019). These promising results, suggest MHY2233 as a synthetic compound with significant potential in anti-senescence research.

Because anti-aging targets are not widely accepted in the drug discovery field, the identification of anti-aging drugs generated from synthetic compounds is still underreported.

### 4.6 Variability in experimental models

Natural antioxidants have shown similar or better effects than their synthetic equivalents in a number of studies. For instance, in multiple investigations, Ginsenoside Rg1 had more effective anti-ageing properties compared to TEMPOL (Chen et al., 2019; Li et al., 2021; Shen et al., 2020; Xu et al., 2019b) (Chen et al., 2020; Li et al., 2021; Shen et al., 2020; Xu TZ. et al., 2019). Reduction of oxidative stress, hindrance of inflammasome activation, decrement of senescence indicators, and improvement of cellular function were demonstrated as the capabilities of both synthetic and natural antioxidants. However, their relative strengths in each of these mechanisms were not equal. For example, while MHY2233 showed higher efficiency in SIRT1 activation compared to resveratrol (Lamichane et al., 2019), Ginsenoside Rg1 was particularly effective in lowering NOX2-mediated ROS generation (Xu et al., 2019b) (Lamichane et al., 2019; Xu TZ. et al., 2019). A more detailed review of the literature illustrates several concerns and issues of the synthetic antioxidants. As noted earlier, there is still uncertainty about the safety of synthetic compound administration as longevity drugs. Despite the therapeutic applications of synthetic antioxidants, there can be considerable variation in their safety profiles. Extended use, especially at high amounts, may be correlated with risks requiring careful monitoring.

### 4.7 Natural antioxidants

By contrast, natural antioxidants usually show fewer negative effects since their biological activities are varied and they have lower toxicity levels. Moreover, future research on synthetic chemicals must include long-term consequences if one is to determine their safe use in clinical environments. An important process that can reduce side effects and increase therapeutic efficacy is the creation of new pharmacological formulations for these medications. For example, by boosting stability and bioavailability, nanocarriers could greatly improve drug delivery. Furthermore, they can provide regulated release, aim at cells or tissues, and help to lower general exposure and possible side effects.

### 4.8 Lack of standardized antioxidant doses

It is necessary to keep in mind that for the optimal anti-aging results, future studies should focus on elucidating the processes behind these disparate effects and consider evaluating potential synergistic combinations of synthetic and natural antioxidants. The studies by Bhayadia et al. (2016), Chen et al. (2023), Khor et al. (2017), Okuni et al. (2022), and Senthil et al. (2016) mentioned the possible benefits of combining synthetic and natural antioxidants, thereby this approach may increase overall antioxidant efficacy (Bhayadia et al., 2016; Chen et al., 2023; Okuni et al., 2022; Senthil et al., 2016; Marashi et al., 2024; Gorjizad et al., 2025; Fathi et al., 2024; Coughlan et al., 2016).

### 4.9 Mitochondrial dysfunction &amp; antioxidants in aging and AD

Antioxidant strategies have attracted much interest in the battle against aging, cellular senescence, and neurodegenerative diseases including Alzheimer’s disease (AD). Dr. Reddy and colleagues, for instance, have looked at how oxidative stress affects AD and aging as well as how antioxidants might be used in treatment.

### 4.10 Role of oxidative stress in aging and neurodegeneration

Reddy and Beal (2008) highlighted the critical role of mitochondrial dysfunction and oxidative stress in aging and AD pathology (Reddy and Beal, 2008). They emphasized that amyloid beta (Aβ) accumulation contributes to mitochondrial impairment, leading to synaptic damage and cognitive decline. This foundational study set the stage for exploring antioxidant-based interventions.

### 4.11 Antioxidant therapeutics in aging and AD

Kandimalla and Reddy (2017) explored the therapeutic potential of neurotransmitter-targeted interventions, underscoring the importance of antioxidant compounds in modulating mitochondrial function and reducing oxidative stress (Kandimalla and Reddy, 2017). Their findings reinforced the concept that mitigating oxidative damage could slow the progression of neurodegenerative disorders.

George et al. (2022) provided a meta-analysis of randomized controlled trials examining cognitive interventions, including antioxidant supplementation, in individuals with dementia. (Saragih et al., 2022). Their systematic review suggested that antioxidant-rich diets and supplementation may help mitigate cognitive decline by reducing oxidative damage and promoting neuronal health.

Sultana et al. (2024) investigated the comparative efficacy of synthetic versus natural antioxidants, demonstrating that naturally derived antioxidants exhibit superior neuroprotective effects. Their research further emphasized the need for targeted antioxidant therapies to prevent senescence-related dysfunctions (Sultana et al., 2024).

### 4.12 Mitochondrial dysfunction and synaptic damage in AD

Reddy (2009) expanded on previous findings by demonstrating that Aβ-induced mitochondrial dysfunction is associated with the phosphorylation of voltage-dependent anion channel 1 (VDAC1), a key regulator of mitochondrial permeability (Reddy, 2013). This study provided further mechanistic insights into how oxidative stress contributes to synaptic degeneration and neuronal loss in AD.

### 4.13 Potential future directions

#### 4.13.1 Challenges in the utilization of these antioxidants in clinical trials

Although preclinical and laboratory studies have shown that both natural and synthetic antioxidants can reduce cellular senescence, there are several obstacles to their therapeutic use in clinical settings. First, target patients or cases should be identified. This could include individuals undergoing chemotherapies that cause senescence or patients displaying the first signs of age-related illnesses. The development of standardized formulations and ideal dosing schedules is necessary, particularly for natural substances because of their diverse pharmacokinetics and bioavailability. Ensuring the long-term safety of antioxidants presents another difficulty. Precise toxicological analyses are essential, especially for synthetic substances with limited clinical data like MHY2233 and TEMPOL. Furthermore, to make it easier to evaluate the effectiveness of antioxidants in clinical trials, reliable markers for senescence and aging-related outcomes should be developed. Future research should consider how combining antioxidant chemicals with alternative therapy approaches, like senolytics or lifestyle changes, may improve efficacy. These problems might be resolved by further clinical research, which would incorporate antioxidant therapies into clinical practice.

These investigations emphasize the role that oxidative stress plays in neurodegeneration and cellular aging. To delay the onset of Alzheimer’s disease and age-related cognitive decline, research from Reddy Lab and related studies supports a multifaceted approach that includes natural antioxidants, mitochondrial stabilizers, and lifestyle changes. Clinical trials evaluating the long-term effectiveness of antioxidant therapies in reducing neurodegenerative development should be the focus of future research.

Including these crucial discoveries in the current systematic review would improve the discussion of antioxidant strategies that target cellular senescence, be consistent with the body of existing research, and address important concerns raised by the reviewers.

### 4.14 Limitations

Notwithstanding the merits of this study, specific limitations must be recognized. The sample size may limit the generalizability of the findings, requiring additional research with larger and more diverse populations. Secondly, any biases in data collection or analysis, such as selection bias or confounding variables, may compromise the validity of the results. Third, the study design may inadequately encompass long-term consequences, highlighting the necessity for longitudinal research. Ultimately, dependence on approaches may restrict the generalizability of the findings to wider clinical or scientific settings. Subsequent research should rectify these shortcomings to improve the robustness and dependability of the results.

## 5 Conclusion

This investigation offers an in-depth comparison of synthetic and natural antioxidant strategies in cellular senescence, emphasizing their potential in mitigating age-related decline and neurodegenerative events. Our findings reinforce the critical role of oxidative stress in aging and neurodegeneration and underscore the therapeutic promise of antioxidants in the fight against these effects. Markedly, studies from leading research groups, including those by Dr. Reddy and colleagues, highlight the value of preserving mitochondrial function, reducing ROS, and promoting cellular resilience to delay aging and tissue dysfunction.

Natural antioxidants often show greater efficacy than synthetic ones due to their enhanced bioavailability, lower toxicity, and the potential for synergistic interactions.

Nonetheless, the effectiveness of antioxidant therapy remains somewhat inconsistent, influenced by parameters such as dosage, method of administration, and individual metabolic characteristics. These difficulties highlight the necessity for additional inquiry.

While synthesizing existing data, we discern a significant deficiency in extensive, controlled clinical studies evaluating the long-term effects of antioxidant therapies, particularly concerning aging and Alzheimer’s disease. Future research should focus on optimizing antioxidant combinations, exploring new substances with multi-targeted effects, and enhancing our understanding of the interrelated molecular processes that govern cellular aging. Building on this foundation, the profession can advance towards formulating effective, evidence-based solutions to foster healthy aging and mitigate the impact of age-related illnesses.

## Data Availability

The original contributions presented in the study are included in the article/Supplementary Material, further inquiries can be directed to the corresponding author.
