# Supplementary material for: Antioxidant strategies against cellular senescence: unveiling the power of synthetic versus natural antioxidants in a systematic review
Source: Front Aging. 2025 May 27;6:1543360. doi: 10.3389/fragi.2025.1543360 (PMC12149200; doi:10.3389/fragi.2025.1543360)
Supplement: Supplementary file 1 [file Table1.docx]

| Reference | Authors | Year | Country | Type of study | Type of antioxidant | Species | Cell type | Mechanism  (discussion, method, result) | Outcome on cellular aging  (result) | Conclusion |
| --- | --- | --- | --- | --- | --- | --- | --- | --- | --- | --- |
| (1) | Bjelica et al. | 2019 | Serbia | In vitro | Synthetic: 5 mM N-acetylcysteine (NAC)  Natural: 100 µM Apocynin (APO) | Human | Peripheral blood mesenchymal stromal cells (PBMSC) | Decreasing lysosomal compartment and activity (SA-β-gal enzyme activity)  Deactivation of the p16-pRB axis  Inhibiting cell cycle arrest at S phase & & enhancing cellular proliferation | NAC and APO prevented hydroxyurea-induced senescence markers, including SA-β-gal activity and p16INK4 expression in PBMSC, with APO showing slightly higher prevention. Together, they also protected against cell cycle arrest in S phase and inhibition of proliferation caused by hydroxyurea. | Antioxidants NAC and APO can prevent premature senescence induced by hydroxyurea in PBMSC, suggesting that ROS mediates hydroxyurea-induced senescence in these cells. |
| (2) | Briganti et al. | 2008 | Italy/Germany | In vitro | Synthetic: 5 mM N-acetylcysteine (NAC)  Natural: 200 µM α-lipoic acid (α-LA), 100 µM α-tocopherol (α-Toc) | Human | Dermal fibroblasts from the foreskin of healthy human donors aged 3 to 6 years | Reducing oxidative stress by decreasing ROS levels & Increasing Gpx, SOD, Catalase  Decreasing lysosomal compartment and activity (SA-β-gal enzyme activity)  Decreasing cytokine secretion (MMP-1)  Decreasing Structural changes | NAC, α-LA, and α-Toc decreased intracellular ROS levels, with α-LA and α-Toc showing slightly higher reduction.  They reduced senescence-associated β-galactosidase (SA-β-gal) activity, with α-LA showing slightly higher reduction.  They lowered matrix metalloproteinase-1 (MMP-1) expression and secretion.  NAC, α-LA, and α-Toc prevented morphological changes associated with senescence, with α-LA supplementation being most effective for rescuing the juvenile fibroblast phenotype.  The antioxidants also partially restored polyunsaturated fatty acid content of cellular membranes, decreased lipid peroxidation products (TBARS), rebalanced activities of antioxidant enzymes (SOD, catalase, glutathione peroxidase), and increased intracellular glutathione levels. | Small molecular antioxidants (NAC, α-LA, α-Toc) effectively protect against PUVA-induced oxidative stress responses underlying fibroblast senescence and photoaging. They reduce ROS levels, protect cellular components from oxidative damage, and restore antioxidant defenses. |
| (3) | Che et al. | 2022 | China | In vitro and in vivo | Synthetic: 150 mg/kg/day N-acetylcysteine (NAC)  Natural: 25 mg/kg/day Spermidine | Human and Mouse | In vitro: Human nucleus pulposus (NP) cells isolated from non-degenerated (grade 1) intervertebral disc tissues  In vivo: Wild-type (WT) C57BL/6J mice, Cdkn2a knockout (p16-/-) mice generated by crossing Cdkn2a heterozygous mice with C57BL/6J mice | Reducing oxidative stress  Reducing DNA Damage  Decreasing Structural changes  Decreasing lysosomal compartment and activity (SA-β-gal enzyme activity)  Deactivation of the p16-pRB axis  Maintaining extracellular matrix (ECM) components (collagen II, aggrecan)  Inhibiting extracellular matrix-degrading enzymes (MMPs, ADAMTS)  inhibiting polyamine catabolizing enzymes, including PAOX, | Both spermidine and NAC supplementation reduced oxidative stress markers in mouse disc tissues, including decreased H2O2 levels and reduced total reactive oxygen species (ROS) levels. They also inhibited DNA damage markers (γH2AX, 8-OH) and inhibited both the H2O2 and ROS accumulation in the disc tissue, with NAC showing slightly higher inhibition.  Both compounds increased total polyamine content in mouse disc tissues, with Spermidine showing higher levels.  Spermidine and NAC supplementation inhibited PAOX (polyamine oxidase) expression in mouse discs, with NAC showing a higher reduction.  They both reduced senescence markers in mouse disc tissues, including decreased p16 levels and reduced β-galactosidase levels, with NAC showing slightly higher reduction.  Regarding effects on extracellular matrix (ECM) components, spermidine protected collagen II and aggrecan levels, and prevented MMP3/9/13 and ADAMTS2 expression. NAC protected collagen II and aggrecan levels and prevented MMP3/9 expression.  Both compounds helped maintain disc height in aged mice, indicating a protective effect against intervertebral disc degeneration. | Overall, the article indicates that spermidine and NAC had similar anti-senescence effects in mouse disc tissues, with slight differences in their impacts on specific extracellular matrix-related genes. |
| (4) | Chen et al. | 2023 | China | In vitro and in vivo | Natural: 20 µM Dendrobine (DEN)  Synthetic: N-acetylcysteine (NAC) | Human and Rat | In vitro: Primary chondrocytes isolated from the knees of 4-week-old Wistar rats, C28I2 human chondrocyte cell line  In vivo: 8-week-old male Wistar rats were used to create an osteoarthritis (OA) model via anterior cruciate ligament transection (ACLT) | Reducing oxidative stress by decreasing ROS levels  Deactivation of the p16-pRB axis  Deactivation of the p53-p21 axis  Decreasing lysosomal compartment and activity (SA-β-gal enzyme activity)  Inhibiting NF-κB pathway activation  Decreasing cytokine secretion (IL-6, TNF-α)  Maintaining extracellular matrix (ECM) components  Inhibiting extracellular matrix-degrading enzymes (MMP13 and ADAMTS5)  Improving mitochondrial function by DEN | Both DEN and NAC were shown to have anti-senescence effects in chondrocytes, though they were not directly compared in most experiments.  DEN inhibited the expression of senescence-associated secretory phenotype (SASP) factors and the senescence phenotype in IL-1β-treated chondrocytes. Specifically, DEN reduced the expression of senescence markers p21 and p16, decreased SA-β-gal positive cells, and inhibited the expression of SASP factors (IL-6, TNF-α).  NAC was used as an ROS inhibitor to further investigate the mechanism of DEN's effects. When NAC was applied in addition to DEN treatment, it further decreased the levels of IL-6, TNF-α, p21, and p16 compared to DEN treatment alone. SA-β-gal staining showed that NAC improved the senescence phenotype compared to DEN-treated chondrocytes.  Both DEN and NAC were found to act through the ROS/NF-κB axis to exert their anti-senescence effects. DEN reduced intracellular ROS levels and inhibited the NF-κB signaling pathway. As a ROS inhibitor, NAC further enhanced these effects when combined with DEN.  Western blot results showed that NAC significantly increased the expression of Col2a1 and ACAN (ECM synthesis markers) and reduced the expression of MMP13 and ADAMTS5 (ECM degradation markers) in DEN-treated chondrocytes. This suggests that NAC enhanced the protective effects of DEN on ECM homeostasis, further supporting the idea that both compounds work through similar mechanisms to protect against osteoarthritis, with NAC potentially having additive or synergistic effects when combined with DEN.  DEN was found to increase the mitochondrial membrane potential, as measured by the JC-1 assay. This indicates that DEN helps maintain mitochondrial health and function. While the article primarily focused on DEN's effects, it did not directly compare DEN and NAC regarding mitochondrial function improvement. | While the article did not directly compare DEN and NAC for most outcomes, it suggested that both compounds have anti-senescence properties in chondrocytes, with NAC potentially having stronger or complementary effects when used in combination with DEN. |
| (5) | Khor et al. | 2017 | Malaysia | In vitro | Synthetic: 1.0 mg/mL N-acetylcysteine (NAC)  Natural: 50 μg/mL Tocotrienol-rich fraction (TRF) & alpha-tocopherol (ATF). TRF consists of α-tocotrienol (ATT; 26.89%), β-tocotrienol (BTT; 3.64%), γ-tocotrienol (GTT; 31.66%), δ-tocotrienol (DTT; 13.66%), and α-tocopherol (ATF; 24.15%)  For combined treatment of TRF and NAC: 25 μg/mL of TRF or ATF with 1.0 mg/mL NAC | Human | Human Skeletal Muscle Myoblasts (HSMM) | Decreasing Structural changes  Decreasing lysosomal compartment and activity (SA-β-gal enzyme activity)  Reducing oxidative stress by decreasing ROS levels and lipid peroxidation  Modulating antioxidant enzyme expression and activity (SOD2, CAT, GPX1)  Maintaining cell viability and reducing apoptosis | TRF and ATF reduced intracellular ROS in senescent myoblasts, as indicated by decreased fluorescence intensity and fewer positively stained cells.  Both treatments significantly diminished intracellular H2O2 generation in presenescent and senescent myoblasts. However, only ATF-treated senescent myoblasts showed significantly lower intracellular superoxide anion levels than untreated controls.  Presenescent cells treated with TRF+NAC and ATF+NAC exhibited significantly lower ROS generation. In senescent myoblasts, only TRF+NAC treatment caused a significant decrease in ROS generation.  NAC treatment alone did not show a similar reduction in free radical generation, suggesting that the antioxidant effects observed were primarily due to TRF and ATF actions rather than NAC.  The combinations of TRF+NAC and ATF+NAC appeared to have enhanced effects in reducing ROS compared to NAC alone, particularly in presenescent and senescent myoblasts. TRF+NAC showed greater efficacy than ATF+NAC in senescent cells specifically.  TRF appeared more effective in modulating antioxidant enzyme activities, particularly in senescent myoblasts, than ATF and NAC alone. The combinations of TRF+NAC and ATF+NAC often showed enhanced effects compared to the individual treatments, suggesting potential synergistic effects in modulating antioxidant enzyme activities.  TRF, ATF, NAC, and their combinations (TRF+NAC and ATF+NAC) all significantly increased the number of viable cells in young, presenescent, and senescent myoblasts compared to their corresponding untreated controls.  Regarding apoptosis, senescent myoblasts showed increases in both early and late apoptotic events compared to young and presenescent cells. Treatment with TRF, ATF, NAC, TRF+NAC, or ATF+NAC significantly reduced the number of early apoptotic cells in senescent myoblasts. However, only TRF, TRF+NAC, and ATF+NAC treatments significantly decreased the number of cells undergoing late apoptotic events in senescent myoblasts. In presenescent myoblasts, early and late apoptotic events were also increased, and these were reduced by TRF+NAC or ATF+NAC treatment. | In summary, TRF generally showed superior anti-senescence effects compared to ATF and NAC, especially in modulating antioxidant defenses. Combinations of TRF or ATF with NAC often showed enhanced effects compared to NAC alone. |
| (6) | Jumnongprakhon et al. | 2021 | Thailand | In vitro | Synthetic: 20 μM N-acetylcysteine (NAC)  Natural: 10-75 μg/mL ethanolic extract of Okra fruit (OKR) | Human | SK-N-SH neuroblastoma cells | Reducing oxidative stress by decreasing ROS levels  Decreasing lysosomal compartment and activity (SA-β-gal enzyme activity)  Inhibiting acetylcholinesterase (AChE) activity  Promoting cell viability | OKR significantly reduced ROS production in H2O2-treated cells in a dose-dependent manner, with 50-75 μg/mL OKR showing similar effects to 20 μM NAC  OKR at 50-75 μg/mL significantly decreased SA-β-gal positive cells compared to H2O2 treatment alone, though less effectively than NAC  OKR at the highest dose (75 μg/mL) inhibited AChE activity similarly to NAC  OKR at 75 μg/mL significantly reversed H2O2-induced decrease in cell viability, with effects comparable to NAC | OKR, particularly at higher doses, showed anti-senescence effects comparable to NAC in H2O2-induced aging of SK-N-SH cells, likely due to its antioxidant properties. OKR may be a potential natural therapeutic agent for alleviating negative responses in aging neurons. |
| (7) | Khan et al. | 2017 | Austria | In vitro | Synthetic: 2 mM N-acetyl cysteine (NAC)  Natural: 1.5 μM Plumericin | Human | Umbilical vein endothelial cells (HUVECs) | Reducing oxidative stress by decreasing ROS levels  Inhibiting NF-κB pathway activation  Decreasing lysosomal compartment and activity (SA-β-gal enzyme activity)  Deactivation of the p16-pRB axis  Deactivation of the p53-p21 axis  Decreasing cytokine secretion (IL-6, IL-8)  Decreasing adhesion molecule expression (E-selectin, ICAM-1)  Maintaining cell proliferation by increasing Ki-67 protein | Both NAC and plumericin prevented TNFα-induced senescence markers, including increased SA-β-gal activity, p16 and p21 expression, and increased Ki-67 expression in HUVECs  NAC and plumericin inhibited TNFα-induced increases in ROS levels and NF-κB activation  The antioxidants prevented TNFα-induced secretion of IL-6 and IL-8 and expression of adhesion molecules E-selectin and ICAM-1  NAC and plumericin maintained HUVEC proliferation during chronic TNFα exposure  Compared to NAC, Plumericin showed a more pronounced effect on reducing ROS production, especially at early time points. However, NAC had a slightly greater impact on rescuing TNFα-impaired cell proliferation and preventing the expression of SASP proteins than plumericin. | Both synthetic (NAC) and natural (plumericin) antioxidants can prevent premature senescence induced by chronic TNFα exposure in endothelial cells by reducing oxidative stress, inhibiting NF-κB signaling, and suppressing the senescence-associated secretory phenotype. |
| (8) | Khatri et al. | 2016 | India | In vitro and in vivo | Synthetic: 10 mM N-acetyl cysteine (NAC)  Natural: 100 mg/kg Curcumin | Mouse | In vitro: Bone marrow stromal cells isolated from young and old mice, Hematopoietic stem and progenitor cells (HSPCs) isolated from mouse bone marrow (including: LSK (Lin-Sca-1+c-kit+) cells, LSKFlk2+ and LSKFlk2- subpopulations)  In vivo: C57Bl/6J mice (CD45.2/Ly5.2), C57Bl/6J SJL mice (CD45.1/Ly5.1), eGFP-expressing C57Bl/6J mice | Reducing oxidative stress by decreasing ROS levels  Increasing glutathione (GSH) levels  Decreasing expression of oxidative stress response genes (catalase, SOD-2, GPX-1, NOX-4)  Decreasing apoptosis  Increasing expression of anti-apoptotic genes (Bcl-2)  Decreasing expression of pro-apoptotic genes (Bax)  Increasing expression of hematopoietic growth factor genes (Flt-3L, SCF)  Improving stromal cell function and hematopoietic reconstitution | Curcumin treatment in vivo significantly reduced ROS levels and increased GSH levels in stromal cells of aged mice. In contrast, NAC treatment in vitro only partially improved the maintenance of hematopoietic stem/progenitor cells by aged stromal cells.  Curcumin reduced the expression of oxidative stress genes and apoptosis in stromal cells in vivo. NAC treatment in vitro reduced ROS levels and apoptosis in old stromal cells, but to a lesser extent than curcumin's in vivo effects.  Curcumin showed more pronounced effects than NAC in rejuvenating aged stromal cell function. In vivo, curcumin improved bone marrow cellularity and hematopoietic reconstitution in aged mice after transplantation. In contrast, NAC treatment in vitro had limited effects on improving stromal cell support of hematopoietic cells.  Curcumin reduced the expression of oxidative stress genes and apoptosis in stromal cells in vivo. NAC treatment in vitro reduced ROS levels and apoptosis in old stromal cells, but to a lesser extent than curcumin's in vivo effects.  Curcumin increased the expression of anti-apoptotic and hematopoietic growth factor genes in stromal cells. Similar gene expression changes were not reported for NAC treatment. | The study found curcumin to have more potent and comprehensive anti-senescence effects on bone marrow stromal cells than NAC, particularly when administered in vivo. However, the authors note that direct head-to-head comparisons were limited since curcumin was tested in vivo while NAC was only evaluated in vitro.. |
| (9) | Lee et al. | 2013 | Republic of Korea | In vitro | Synthetic: N-acetylcysteine (NAC)  Natural: Apocynin | Human | Breast cancer (MCF-7) and colorectal cancer (HCT116) cells | Reducing oxidative stress by decreasing ROS levels with both NAC & Apocynin  Decreasing lysosomal compartment and activity (SA-β-gal enzyme activity) by both NAC & Apocynin  Deactivation of the p53-p21 axis by both NAC & Apocynin  Inhibiting NADPH oxidase activation by Apocynin | NAC and apocynin suppressed coumestrol-induced reactive oxygen species (ROS) generation in MCF-7 and HCT116 cells, as indicated by decreased fluorescence intensity during flow cytometry analysis. Cell-treatment with either NAC or apocynin reduced senescence-associated β-galactosidase (SA-β-gal) activity compared to cells treated with coumestrol alone.  Both NAC and apocynin decreased p53 protein expression induced by coumestrol treatment.  Apocynin specifically inhibits NADPH oxidase, which was shown to be involved in coumestrol-induced ROS generation and subsequent senescence.  NAC and apocynin both inhibited the senescence phenotype caused by coumestrol treatment in breast and colorectal cancer cells. | While both compounds showed similar anti-senescence effects, the article did not directly compare their relative efficacies. Instead, it presented them as two different approaches (ROS scavenging for NAC and NADPH oxidase inhibition for apocynin) to achieve similar outcomes in preventing coumestrol-induced cellular senescence. |
| (10) | Lee et al. | 2014 | South Korea | In vitro | Synthetic: N-acetyl-L-cysteine (NAC)  Natural: Apocynin | Human | IMR-90 lung fibroblasts and HCT116 colon cancer cells | Reducing oxidative stress by decreasing ROS levels  Deactivation of the p53-p21 axis  Decreasing lysosomal compartment and activity (SA-β-gal enzyme activity)  Inhibiting NADPH oxidase activity | Both NAC and apocynin reduced ROS generation induced by PLD2 knockdown  Both NAC and apocynin suppressed the appearance of senescence markers, including SA-β-gal activity  Both NAC and apocynin inhibited the upregulation of p53 and p21 expression caused by PLD2 downregulation | NAC and apocynin prevented cellular senescence induced by PLD2 downregulation by inhibiting ROS production, suggesting ROS mediates PLD2 knockdown-induced senescence in these cells. |
| (11) | Li et al. | 2016 | China | In vitro and in vivo | Synthetic antioxidants: N-acetylcysteine (NAC, 100 μmol/L), Metformin (Met, 0.1 mmol/L), Rapamycin (Rap, 10 nmol/L)  Natural antioxidants: Vitamin C (VC, 280 μmol/L), Vitamin E (VE, 10 μmol/L), (-)-Epigallocatechin gallate (EGCG, 2 μmol/L), Resveratrol (Res, 2 μmol/L) | Human and Mouse | In vitro: WRN gene knockout human embryonic stem cell (ESC) clones (referred to as WRN-/- ESCs), Wild-type human ESCs (WT ESCs), Mesenchymal stem cells (MSCs) derived from both WRN-/- ESCs and WT ESCs  In vivo: Luciferase-expressing WRN-/- MSCs were implanted into immunodeficient mice to assess cell viability in vivo | Reducing oxidative stress by decreasing reactive oxygen species (ROS) levels with Vitamin C  Decreasing lysosomal compartment and activity (senescence-associated β-galactosidase staining)  Alleviating telomere attrition by Vitamin C  Decreasing cytokine secretion (IL-6, IL-8) by Vitamin C  Restoring heterochromatin architecture by Vitamin C  Reactivating cell proliferation potential by increasing Ki-67 protein with Vitamin C  Upregulating the expression of genes involved in cell cycle, DNA replication, and repair  Deactivation of the p16-pRB axis  Downregulating pro-senescence pathways genes (p53, FoxO, HIF-1), suggesting Deactivation of p53-p21 axis | In the initial screening, VC showed the most potent effect among all tested compounds in reducing senescence-associated β-galactosidase (SA-β-gal) positive cells in WS MSCs, while NAC demonstrated only mild activity. VC was effective at physiological concentrations starting from as low as 10 μmol/L, whereas NAC required higher concentrations to show an effect. VE, EGCG, Met, Rap, and Res showed varying degrees of effectiveness, but were generally less potent than VC.  Vitamin C restored proliferation, reduced ROS levels, alleviated telomere shortening, and decreased secretion of inflammatory factors  Vitamin C restored heterochromatin organization and nuclear lamina structure,  down-regulated the expression of aging markers, including p16^Ink4a and GATA4, in WS MSCs.  Vitamin C treatment reactivated the expression of genes related to cell cycle, DNA replication, and repair that are repressed in WS MSCs | Vitamin C was identified as the most effective compound for alleviating premature aging phenotypes in WS MSCs compared to other tested antioxidants and compounds. |
| (12) | Moon et al. | 2009 | Republic of Korea | In vitro | Synthetic: N-acetylcysteine (NAC)  Natural: Clitocybins B and C isolated from the mushroom Clitocybe aurantiaca | Human | IMR-90 lung fibroblast cells | Reducing oxidative stress by decreasing ROS levels  Inhibiting NF-κB pathway activation by preventing IκB-α and IκB-β degradation  Decreasing caspase 3 and 9 activation  Preventing cytochrome c release from mitochondria  Decreasing lysosomal compartment and activity (SA-β-gal enzyme activity)  Promoting cell viability | Clitocybins and NAC reduced intracellular reactive oxygen species (ROS) levels in H2O2-treated cells, with Clitocybin C showing slightly higher ROS scavenging ability than Clitocybins A and B. Still, all Clitocybina were less effective than NAC.  Clitocybins and NAC inhibited H2O2-induced cell death, with NAC showing a more complete protective effect than Clitocybins.  Clitocybins and NAC prevented H2O2-mediated increases in senescence-associated β-galactosidase (SA-β-gal) positive cells, indicating an anti-senescence effect.  Both types of antioxidants reduced H2O2-induced caspase-3 and caspase-9 activation and cytochrome c release from mitochondria, suggesting similar mechanisms of action in preventing apoptosis.  Clitocybins and NAC both inhibited NF-κB activation by preventing IκB-α and IκB-β degradation in response to H2O2 treatment.  Clitocybina maintained a higher cell density compared to the H2O2-treated control. However, the cell density in the clitocybin-treated groups was not as high as in the NAC-treated group. | Both synthetic (NAC) and natural (clitocybins). While Clitocybina and NAC had similar overall anti-senescence effects, there were some slight differences in their efficacy, with NAC generally showing more potent effects. However, the authors suggested that Clitocybina represents novel natural compounds with anti-senescence properties comparable to the well-known synthetic antioxidant NAC. |
| (13) | Okuni et al. | 2022 | Japan | In vitro and in vivo | Synthetic: 0, 5, or 7.5 mM N-acetyl cysteine (NAC)  Natural: 0, 100, 200 μM α-tocopherol | Human and Mouse | In vitro: Pancreatic cancer cells (Panc1, CFPAC1, MIAPaCa2, BxPC3, Capan2), T-cell malignant cell line (MOLT4)  In vivo: CFPAC1 cells were used for xenograft studies in nude mice. | Reducing oxidative stress by decreasing ROS levels  Promoting cell proliferation  Decreasing mitochondrial lipid peroxidation  Maintaining mitochondrial membrane potential  Decreasing lysosomal compartment and activity (SA-β-gal enzyme activity) by α-Tocopherol  Reducing the downregulation of FOXM1 expression by α-Tocopherol | NAC effectively prevented growth inhibition by romidepsin, while α-tocopherol prevented growth inhibition induced by tamoxifen. The combination of NAC and α-tocopherol was more effective in reducing reactive oxygen species (ROS) generation and preventing growth inhibition induced by romidepsin plus tamoxifen compared to either antioxidant alone.  NAC was more effective at inhibiting ROS accumulation at early time points (9-12 hours after treatment), while α-tocopherol showed greater efficacy in preventing lipid peroxidation in mitochondria. Both antioxidants helped maintain mitochondrial membrane potential when used in combination.  α-Tocopherol effectively prevented siomycin A-induced senescence and growth inhibition by reducing senescence-associated β-galactosidase activity in untreated and siomycin A-treated cells. This suggests α-tocopherol may have a more potent effect on FOXM1-mediated senescence pathways. | Both synthetic (NAC) and natural (α-tocopherol) antioxidants can prevent premature senescence induced by romidepsin and tamoxifen in pancreatic cancer cells by reducing oxidative stress, protecting mitochondria, and maintaining FOXM1 expression (by α-tocopherol). This suggests that antioxidants may counteract the anti-cancer effects of these drugs by inhibiting senescence induction. |
| (14) | Pan et al. | 2022 | China | In vitro | Synthetic: 5 mM N-acetylcysteine (NAC)  Natural: 1 mM Acetyl-L-carnitine (ALC) | Human | Adipose-derived stem cells (ADSCs) | Reducing oxidative stress by decreasing ROS levels and increasing antioxidant enzyme expression (SOD1, catalase)  Modulating autophagy by regulating Bcl-2-Beclin1 and Bcl-2-Bax complex dissociation  Inhibiting apoptosis  Deactivation of the p16-pRB axis  Maintaining stem cell marker expression (OCT4, NANOG, SOX2)  Decreasing lysosomal compartment and activity (SA-β-gal enzyme activity)  Maintaining cell proliferation by increasing Ki-67 protein & EdU incorporation | Both NAC and ALC reduced intracellular ROS levels in serum-starved ADSCs, with NAC showing a greater reduction  ALC maintained autophagy levels while NAC inhibited autophagy. This suggests ALC and NAC have different mechanisms of action, with ALC's protective effects partially dependent on maintaining autophagy.  Both ALC and NAC inhibited senescence markers in starved ADSCs, as evidenced by decreased β-galactosidase activity and reduced expression of p16. However, ALC (1 mM) showed slightly better effects in preventing senescence than NAC.  Both ALC and NAC reduced the apoptosis rate.  ALC maintained expression of stem cell markers (OCT4, NANOG, SOX2) better than NAC in serum-starved conditions, indicating ALC may be more effective at preserving stemness.  ALC improved cell proliferation more than NAC in serum-starved ADSCs, as measured by increased Ki-67 expression & EdU incorporation | While both compounds protected against senescence, ALC's effects appeared to rely on reducing ROS and modulating autophagy, whereas NAC's effects were primarily through ROS reduction. This suggests that ALC may have a more comprehensive anti-senescence mechanism than NAC in this model. |
| (15) | Park and Kim | 2012 | Korea | In vitro | Synthetic: 50 μM N-acetylcysteine (NAC)  Natural: 100 µM L-arginine, 100 µM sepiapterin | Human | Human trabecular meshwork cells (HTMC) | Reducing oxidative stress by decreasing reactive oxygen species levels  Increasing nitric oxide production  Decreasing lysosomal compartment and activity (SA-β-gal enzyme activity)  Maintaining cell survival | Regarding oxidative stress, L-arginine, sepiapterin, and NAC all significantly decreased AGE-induced superoxide production compared to non-exposed trabecular meshwork cells. However, only NAC abolished the AGE-induced increase in reactive oxygen species generation, while L-arginine and sepiapterin did not have this effect.  The antioxidants increased nitric oxide production, which AGE reduced.  Regarding cellular senescence, L-arginine, sepiapterin, and NAC all significantly decreased the AGE-induced senescence of trabecular meshwork cells, as measured by senescence-associated β-galactosidase staining. The anti-senescence effects were observed at 50 μg/mL and 100 μg/mL AGE concentrations.  NAC, L-arginine, and sepiapterin maintained cell survival that was reduced by AGE exposure | Both synthetic (NAC) and natural (L-arginine, sepiapterin) antioxidants can prevent AGE-induced oxidative stress and cellular senescence in human trabecular meshwork cells by reducing reactive oxygen species, increasing nitric oxide production, and suppressing senescence markers. NAC appeared to have the most comprehensive antioxidant effects by reducing superoxide and overall reactive oxygen species levels. |
| (16) | Ren et al. | 2022 | China | In vitro and in vivo | Synthetic: 10 μM N-acetylcysteine (NAC)  Natural: 1000 μM Nicotinamide mononucleotide (NMN) | Human and mouse | In vitro: ARPE-19 cells (adult human retinal pigment epithelial cell line)  In vivo: C57BL/6J male mice (6-8 weeks old) | Reducing oxidative stress by decreasing ROS levels  Decreasing DNA damage (reduced γ-H2AX foci)  Improving mitochondrial function (restored mitochondrial membrane potential and ATP levels)  Decreasing lysosomal compartment and activity (reduced SA-β-gal staining)  Deactivation of p16-pRB and p53-p21 axes (reduced p16 and p21 expression)  Maintaining NAD+ levels & Increasing SIRT1 expression  Modulating AMPK and Akt/mTOR signaling pathways  Preserving RPE morphology and barrier function  Reducing subretinal inflammation (decreased CD11b+ and F4/80+ cell infiltration) | NMN showed more substantial anti-senescence effects than NAC in sodium iodate-induced RPE senescence. NMN more effectively reduced SA-β-gal staining, p16 and p21 expression, DNA damage, and ROS levels. NMN also better preserved mitochondrial function, NAD+ levels, and RPE morphology.  NAC significantly decreased reactive oxygen species (ROS) levels in sodium iodate-treated cells but failed to prevent the RPE cells from entering senescence. In contrast, NMN treatment strongly ameliorated RPE senescence through multiple mechanisms.  NMN more effectively reduced senescence markers compared to NAC, including decreasing senescence-associated β-galactosidase (SA-β-gal) staining, p16 and p21 expression, and DNA damage (by reducing γ-H2AX-positive cells).  NMN showed superior effects in preserving mitochondrial function compared to NAC. While NAC only minimally alleviated cellular senescence, NMN restored mitochondrial membrane potential and ATP levels in senescent RPE cells.  NMN supplementation, but not NAC, maintained NAD+ levels in the cells and tissues. This allowed NMN to activate sirtuin 1 (SIRT1) signaling, crucial for its anti-senescence effects.  In vivo, NMN treatment reduced RPE dysfunction and senescence-associated retinal inflammation, while NAC did not observe such effects.  NAC only minimally alleviated cellular senescence, while NMN strongly ameliorated RPE senescence through multiple mechanisms, including reducing DNA damage, maintaining mitochondrial function, and modulating key signaling pathways. NMN's effects were dependent on Sirt1 signaling. | NMN supplementation effectively ameliorates RPE cellular senescence and senescence-associated retinal inflammation by restoring NAD+ levels and improving mitochondrial function. NMN shows greater potential than NAC as a therapeutic strategy for age-related macular degeneration from the perspective of cellular senescence. |
| (17) | Senthil et al. | 2016 | Taiwan | In vitro and in vivo | Synthetic: 100 μM N-acetylcysteine (NAC)  Natural: 10 μM Antcin M (ANM) and and 5 μM Resveratrol (RES) | Human and C. elegans | In vitro: Human normal dermal fibroblasts (HNDFs, CCD966SK cell line), Human umbilical vein endothelial cells (HUVECs)  In vivo: Caenorhabditis elegans (C. elegans) wild-type Bristol N2 strain | Reducing oxidative stress by decreasing ROS levels  Activating Nrf2-dependent antioxidant genes (HO-1, NQO-1)  Decreasing lysosomal compartment and activity (SA-β-gal enzyme activity)  Deactivation of p16-pRB and p53-p21 axes  Inhibiting p53 and FoxO1 acetylation  Upregulating SIRT1 expression and activity  Maintaining cell cycle progression and proliferation  Inhibiting JNK/SAPK and p38 MAPK activation | ANM & NAC effectively rescued high glucose-induced reduction in cell cycle regulatory proteins (cyclins, CDKs) and promoted cell proliferation.  ANM, NAC & RES inhibited high glucose-induced reactive oxygen species (ROS) generation, with RES & NAC having a greater effect.  Both ANM and NAC activate the Nrf2 antioxidant pathway in HNDFs through different mechanisms. Unlike NAC, ANM did not directly scavenge free radicals. Instead, ANM upregulated antioxidant genes like HO-1 and NQO-1 by enhancing Nrf2 transcriptional activity. ANM increased nuclear translocation of Nrf2 and ARE-luciferase activity to a similar degree as NAC. However, ANM's effects were mediated through AKT and ERK1/2 signalling activation, while NAC likely acts as a direct antioxidant.  ANM and NAC decreased SA-β-gal activity.  ANM & NAC inhibited p16 and p21 expression.  Both compounds prevented p53 and FoxO1 acetylation.  ANM was more effective at maintaining cell cycle progression and proliferation than NAC in HNDFs.  ANM is partially protected against the HG-induced reduction in cell proliferation in SIRT-1-silenced cells. ANM partially inhibited the HG-induced decrease in cell proliferation in Nrf2 knockdown cells, but failed to rescue proliferation when both Nrf2 and SIRT-1 were knocked down. Complete protection against HG-induced reduction in proliferation was achieved by combining ANM with either NAC or resveratrol.  ANM & NAC inhibited stress-activated kinases  ANM extended C. elegans lifespan under normal and high glucose conditions, similar to RES | The natural antioxidant anthocyanin M exhibits more potent anti-senescence effects than synthetic NAC in hyperglycemia-induced cellular senescence models. ANM acts through multiple mechanisms, including Nrf2 activation, SIRT1 upregulation, and inhibition of stress signaling pathways, to prevent premature senescence more effectively than NAC. |
| (18) | Shiwakoti et al. | 2022 | Republic of Korea | In vitro and ex vivo | Synthetic: 10 mM N-acetylcysteine (NAC)  Natural: 10 μM Apocynin (APO), 10 μM Resveratrol (RES) | Pig | In vitro: Porcine coronary artery endothelial cells (PCAECs)  Ex vivo: Porcine coronary artery (PCA) rings | Reducing oxidative stress by decreasing ROS levels  Inhibiting NADPH oxidase expression and activity  Increasing Sirt1 expression  Increasing eNOS expression  Decreasing lysosomal compartment and activity (SA-β-gal enzyme activity)  Deactivation of p53-p21 axis  Deactivation of the p16-pRB axis  Maintaining endothelium-dependent vasorelaxation | APO, RES, and NAC decreased NPs-induced reactive oxygen species (ROS) formation in PCAECs and PCA rings, with similar efficacy.  NAC, APO, and RES prevented nanoplastics-induced increase in senescence-associated β-galactosidase activity in both porcine coronary artery endothelial cells (PCAECs) and porcine coronary artery (PCA) rings.  NAC, APO, and RES inhibited nanoplastics-induced upregulation of p53, p21, and p16 expression  NAC, APO and RES restored cell proliferation impaired by nanoplastics exposure  The antioxidants prevented nanoplastics-induced endothelial dysfunction by maintaining endothelium-dependent vasorelaxation  APO, RES, and NAC reduced NPs-induced expression of NADPH oxidase subunits Nox2 and p22phox in PCAECs, with similar efficacy.  APO, RES increased Sirt1 expression to a similar extent in NPs-treated cells, Slightly higher than NAC.  In terms of improving NPs-induced endothelial dysfunction, RES showed the most pronounced effect in restoring endothelium-dependent relaxation in PCA rings (99.77% relaxation), followed by APO (89.16%) and NAC (88.8%), compared to NPs treatment alone (68.86%). | Both synthetic (NAC) and natural (APO, RES) antioxidants can prevent premature senescence induced by nanoplastics exposure in endothelial cells by reducing oxidative stress, inhibiting NADPH oxidase, increasing Sirt1/eNOS expression, and suppressing senescence markers. RES showed some enhanced effects compared to NAC and APO. |
| (19) | Wang et al. | 2018 | Taiwan/USA | In vivo and in vitro | Synthetic: 5 mM N-acetylcysteine (NAC)  Natural: 400 μg/mL caffeine in drinking water | Human, Mouse and Golden hamster | In vitro: Human aortic endothelial cells (HAECs), Chinese hamster ovary (CHO) cells  In vivo: C57B6/J mice, LOX-1 knockout mice (LOX-1-/-), Golden Syrian hamsters | Reducing oxidative stress by decreasing ROS levels  Inhibiting DNA damage response (DDR) pathway activation  Decreasing lysosomal compartment and activity (SA-β-gal enzyme activity)  Deactivation of p53-p21 axis | Both NAC and caffeine prevented L5-induced increases in SA-β-gal activity, γH2AX foci formation, p53 and p21 expression in mouse aortic endothelium and cultured human aortic endothelial cells  NAC and caffeine inhibited L5-induced mitochondrial ROS production and activation of the ATM/Chk2/p53 DDR pathway  both NAC and caffeine reduced L5-induced oxidative stress markers, including 3-nitrotyrosine levels, in mouse aortic tissues  Both caffeine and NAC were shown to attenuate L5-induced senescence effects, such as blocking L5-induced activation of DDR proteins (ATM, p-Chk2 T68, TP53, and p21) in Western blot analysis | Both synthetic (NAC) and natural (caffeine) antioxidants can prevent premature vascular endothelial senescence induced by electronegative LDL (L5) by reducing oxidative stress, inhibiting DNA damage signaling, and maintaining telomerase function. |
| (20) | Wang et al. | 2019 | China | In vitro and in vivo | Natural: Cordycepin (200 μM in vitro, 60 mg/kg in vivo)  Synthetic: N-acetylcysteine (NAC) | Human, Rat and mouse | In vitro: Primary human fibroblasts (obtained from foreskin tissues), Human immortalized keratinocytes (HaCaT cells)  In vivo: Sprague-Dawley (SD) rats, C57BL/6J mice | Reducing oxidative stress by decreasing ROS levels and increasing antioxidant enzymes (SOD1, SOD2, GPX-1, Catalase) by Cordycepin & NAC  Decreasing DNA damage (reduced γ-H2AX foci) by Cordycepin  Decreasing lysosomal compartment and activity (reduced SA-β-gal activity) by Cordycepin & NAC  Deactivation of p16-pRB and p53-p21 axes (reduced p16 and p21 expression) by Cordycepin  Decreasing cytokine secretion (reduced IL-1β, IL-6, TNF-α) by Cordycepin  Activating AMPK and NRF2 pathways by Cordycepin  Promoting autophagic degradation of Keap1 by Cordycepin  Maintaining cell proliferation (increased Ki-67-positive cells) by by Cordycepin | Cordycepin reduced senescence markers including SA-β-gal activity, p16/p21 expression, and SASP factors in vitro and in radiation-induced skin, intestinal and oral ulcer models  Cordycepin maintained proliferation and reduced apoptosis of cells after radiation exposure  Cordycepin prevented radiation-induced loss of proliferative basal epithelial stem cells in vivo  NAC treatment reduced senescence markers in fibroblasts in vitro, including decreased SA-β-gal activity and p16/p21 expression | Cordycepin prevents radiation-induced ulcers by inhibiting cellular senescence through activation of the AMPK-NRF2 pathway. Both cordycepin and the synthetic antioxidant NAC show protective effects against radiation-induced senescence markers in vitro. Cordycepin demonstrates significant efficacy in preventing radiation-induced ulcers in vivo, suggesting that natural compounds like cordycepin may be promising for mitigating radiation-induced tissue damage and senescence. |
| (21) | Wang et al. | 2022 | China | In vivo and in vitro | Natural: 1-100 μM of 2,3,4',5-tetrahydroxystilbene-2-O-β-d-glycoside (TSG)  Synthetic: 2 mM of N-acetyl-L-cysteine (NAC) | Human and Mouse | In vitro: Human aortic endothelial cells (HAECs)  In vivo: 12-month-old male LDL receptor-deficient (LDLr-/-) mice | Activating PGC-1α-mediated signaling cascades  Increasing telomerase activity and TERT expression  Reducing oxidative stress by decreasing ROS levels  Improving mitochondrial function  Increasing antioxidant enzyme expression (Mn-SOD, CAT)  Decreasing lysosomal compartment and activity (SA-β-gal)  Deactivation of p16-pRB and p53-p21 axes | TSG showed dose-dependent effects in reducing senescence markers, with 10 μM and 100 μM doses being most effective. NAC at 2 mM was used as a positive control and showed similar anti-senescence effects to the higher doses of TSG.  In ox-LDL-treated human aortic endothelial cells (HAECs), both TSG and NAC significantly alleviated senescence markers, including decreased SA-β-gal positive cells and reduced expression of aging-related genes γ-H2AX, p53, p21, and p16.  Both TSG and NAC decreased intracellular ROS levels and lipid peroxidation (MDA production) induced by ox-LDL in HAECs. They also reversed the ox-LDL-induced decreases in cellular SOD and CAT activities.  both TSG and NAC demonstrated protective effects against ox-LDL-induced endothelial senescence by increasing telomerase activity, improving mitochondrial function, reducing oxidative stress, and likely activating the PGC-1α pathway. TSG, particularly at higher concentration (100 μM), showed slightly more pronounced effects than NAC in most of these parameters. | TSG protects against vascular senescence and atherosclerosis by activating PGC-1α-mediated signaling cascades, which improve telomere function, reduce oxidative stress, and enhance mitochondrial function. TSG showed similar (or superior in some cases) anti-senescence effects compared to NAC in endothelial cells. |
| (22) | Yang et al. | 2018 | China | In vitro | Synthetic: 10 mM N-acetylcysteine (NAC)  Natural: 200 μM ascorbic acid (AA) | Rat | Bone marrow mesenchymal stem cells (BMSCs) | Reducing oxidative stress by decreasing ROS levels  Inhibiting AKT/mTOR signaling pathway activation  Decreasing lysosomal compartment and activity (SA-β-gal enzyme activity)  Deactivation of p16-pRB axis  Maintaining cell proliferation | Both AA and NAC reduced D-galactose-induced increases in ROS levels and activation of AKT/mTOR signaling in BMSCs  AA and NAC decreased the number of SA-β-gal positive cells and expression of p16 induced by D-galactose exposure  AA and NAC maintained BMSC proliferation during D-galactose exposure | Both natural (AA) and synthetic (NAC) antioxidants can prevent premature senescence induced by D-galactose in BMSCs by reducing oxidative stress and inhibiting AKT/mTOR signaling pathway activation |
| (23) | Yu et al. | 2011 | Korea | In vitro | Synthetic: 10 mM N-acetylcysteine (NAC)  Natural: 1 μM Rotenone, 100 μM Apocynin | Human | Umbilical vein endothelial cells (HUVEC) | Reducing oxidative stress by decreasing ROS levels  Increasing nitric oxide production  Maintaining cell proliferation | The study found that all three antioxidants - NAC, rotenone, and apocynin - ameliorated indoxyl sulfate (IS)-induced changes in human umbilical vein endothelial cell (HUVEC) proliferation, senescence, and nitric oxide production. Specifically, pretreatment with NAC, rotenone, or apocynin prevented the IS-induced inhibition of cell proliferation, increase in cell senescence, and decrease in nitric oxide production at 48 hours. | The similar effects of these antioxidants with different mechanisms of action suggest that IS-induced endothelial dysfunction, expressed as decreased NO production, inhibited cell proliferation, and increased senescence, may be explained by IS-induced oxidative stress through activation of both NADPH oxidase and the mitochondrial respiratory chain complex. The study did not indicate that any one of these antioxidants was superior to the others in preventing IS-induced cellular senescence. |
| (24) | Zhou et al. | 2020 | China | In vivo | Synthetic: 1 mg/ml N-acetylcysteine (NAC)  Natural: 17β-estradiol (E2) at a dose of 10 µg/kg body weight | Mouse | 3-week-old female C57/BL6J mice | Reducing oxidative stress by decreasing ROS levels and increasing antioxidant enzyme activity  Reducing DNA damage by decreasing γ-H2AX and 8-OHdG levels  Decreasing lysosomal compartment and activity (SA-β-gal enzyme activity)  Deactivation of p16-pRB axis  Decreasing cytokine secretion (IL-1α, IL-1β, MMP13, TNF-α, MMP3)  Maintaining bone mineral density and microarchitecture  Inhibiting osteoclastic bone resorption | Both NAC and E2 supplementation reduced oxidative stress markers in ovariectomized mice, including decreased ROS levels, increased total antioxidant capacity, and increased SOD2 expression  NAC and E2 decreased DNA damage markers γ-H2AX and 8-OHdG in osteocytes  Both compounds reduced senescence markers in osteocytes, including decreased p16 and β-galactosidase levels. The figures of the article suggest that E2 exhibited a marginally superior effect in reducing β-galactosidase levels compared to NAC.  NAC and E2 inhibited the senescence-associated secretory phenotype by reducing expression of IL-1α, IL-1β, MMP13, TNF-α, and MMP3. While both compounds showed significant effects in reducing these SASP markers, the figures of the article suggest that E2 exhibited a slightly superior efficacy compared to NAC in this aspect of cellular senescence. inhibition.  Both treatments maintained bone mineral density, trabecular bone volume, and inhibited osteoclast activity in ovariectomized mice | Both synthetic (NAC) and natural (E2) antioxidants can prevent ovariectomy-induced bone loss and cellular senescence in osteocytes by reducing oxidative stress, DNA damage, and the senescence-associated secretory phenotype.  The article did not report any significant differences between E2 and NAC in their anti-senescence effects, suggesting they had similar potency in preventing cellular senescence in this model of postmenopausal osteoporosis. |
| (25) | Bhayadia et al. | 2016 | Germany | Ex vivo and in vitro | Synthetic: 1 mM TEMPOL  Natural: 0.1 mM apocynin | Mouse | In vitro: Primary endothelial cells isolated from mouse lungs  Ex vivo: Aortic rings isolated from Young (3-4 months old) and aged (24-28 months old) C57Bl/6 mice, Aortic rings isolated from Terc-/- mice (G1 and G3 generations) and Terc+/+ littermates | Reducing oxidative stress by decreasing ROS levels  Improving endothelium-dependent vasodilation | Combination of TEMPOL and apocynin significantly improved endothelium-dependent vasodilation in aortic rings from aged wildtype and (Terc-/-) G3 mice with short telomeres compared to untreated controls. This improvement was associated with a reduction in reactive oxygen species (ROS) levels, particularly superoxide (O2-) abundance.  Treatment with TEMPOL or apocynin alone did not improve endothelium-dependent vasodilation in their model. This suggests that the combination of both antioxidants was necessary to achieve the observed beneficial effects on endothelial function.  The antioxidant treatment restored endothelial function without altering the underlying senescence phenotype. | These outcomes indicate that oxidative stress is a key mediator of endothelial dysfunction associated with both age-related and telomere-dependent models of vascular aging, and that antioxidant treatment can restore endothelial function impaired by cellular senescence. |
| (26) | Chen et al. | 2019 | China | In vivo | Synthetic: Tempol (50 mg/kg)  Natural: Ginsenoside Rg1 (5 and 10 mg/kg), Apocynin (50 mg/kg) | Mouse | Neurons in brain cortex and hippocampus of Senescence-accelerated mouse prone 8 (SAMP8) mice as the experimental model of accelerated aging and Senescence-accelerated resistant mouse 1 (SAMR1) mice as the normal aging control group | Reducing oxidative stress by decreasing NOX2-mediated ROS levels and decreasing expressions of NOX2, p22phox and p47phox  Inhibiting NLRP1 inflammasome activation & decreasing expressions of NLRP1, ASC, Caspase-1 and IL-1β  Alleviating neuronal damage and improving neuronal morphology  Improving learning, memory and exploratory behavior | Tempol, Apocynin and Ginsenoside Rg1 all significantly decreased ROS levels in brain tissues of SAMP8 mice. Ginsenoside Rg1 (10 mg/kg) reduced ROS levels more than other antioxidants.  Ginsenoside Rg1 (10 mg/kg) treatment significantly reduced expressions of NOX2, p22phox and p47phox, while Tempol and Apocynin had more limited effects  Rg1, tempol, and apocynin treatment all significantly decreased the expressions of NLRP1 inflammasome components NLRP1, ASC, Caspase-1, and IL-1β in the cortex and hippocampus of SAMP8 mice. Ginsenoside Rg1 at 10 mg/kg showed more pronounced effects in reducing these inflammasome markers compared to tempol, apocynin, and the lower 5 mg/kg Rg1 dose.  Rg1, Apocynin and Tempol all alleviated aging-related neuronal damage in cortex and hippocampus, as assessed by H&E and Nissl staining. Rg1 at 10 mg/kg showed the most pronounced protective effect. | Ginsenoside Rg1 showed superior anti-aging effects compared to Tempol and Apocynin in SAMP8 mice, likely due to its ability to inhibit both NOX2-mediated ROS generation and NLRP1 inflammasome activation |
| (27) | Dai et al. | 2022 | China | In vitro and in vivo | Synthetic: 20 μM Setanaxib  Natural: 100 μM Apocynin | Rat | In vitro: Rat annulus fibrosus (AF) primary cells  in vivo: Rat lumbar disc cells (for the persistent lumbar degeneration model), Rat caudal disc cells (for the caudal disc puncture model) | Reducing oxidative stress by inhibiting NADPH oxidase 4 (NOX4) & maintaining extracellular matrix (ECM) components by inhibiting NOX4  Delaying senescence onset by inhibiting phosphorylation of mitogen-activated protein kinases (MAPK)  Decreasing lysosomal compartment and activity (SA-β-gal enzyme activity)  Deactivation of p53-p21-Rb and p16-Rb axes  Decreasing cytokine secretion (IL-1β, TNF-α) | Both Setanaxib and Apocynin prevented AOPPs-induced senescence markers including increased SA-β-gal activity, p53, p21, and p16 expression in AF cells  The antioxidants inhibited AOPPs-induced increases in IL-1β and TNF-α secretion  Setanaxib and Apocynin maintained AF cell proliferation and prevented G1 phase arrest during AOPPs exposure  Both compounds reduced AOPPs-induced phosphorylation of MAPK pathways (p38, ERK, and JNK) | Both synthetic (Setanaxib) and natural (Apocynin) antioxidants can attenuate AF cell senescence and delay intervertebral disc degeneration by inhibiting the AOPPs/NOX4 pathway. This reduces oxidative stress, inhibits MAPK signaling, and suppresses the senescence-associated secretory phenotype. |
| (28) | Lamichane et al. | 2019 | South Korea | In vitro | Synthetic: 10 nM MHY2233  Natural: 100 nM Resveratrol | Human | Endothelial progenitor cells (EPCs) | Increasing SIRT1 expression and deacetylase activity  Deactivating p53-p21 axis  Deactivating p16-pRB axis  Decreasing senescence-associated β-galactosidase (SA-β-gal) activity  Decreasing senescence-associated secretory phenotype (SASP) factors (IL-6, IL-8, IL-1α, IL-1β)  Reducing oxidative stress by decreasing cellular reactive oxygen species (ROS) levels  Increasing eNOS phosphorylation  Enhancing cell proliferation by increasing cyclin D1 and cyclin E expression  Promoting cell cycle progression  Enhancing cell migration  Increasing angiogenic capacity  Decreasing apoptosis | MHY2233 demonstrated greater potency than resveratrol in activating SIRT1 and increasing its deacetylase activity, even at lower concentrations.  Both MHY2233 and resveratrol decreased senescence markers like SA-β-gal activity, p16, p21, and p53 expression, but MHY2233 showed stronger effects.  MHY2233 and resveratrol both reduced oxidative stress and cellular ROS levels, with MHY2233 showing a more pronounced effect.  Both compounds enhanced cell proliferation, migration, and angiogenic capacity of senescent EPCs (by increasing cyclin D1 and cyclin E expression), but MHY2233 demonstrated greater potency.  MHY2233 more effectively increased eNOS phosphorylation compared to resveratrol and downregulated senescence-associated secretory phenotype (SASP) factors (IL-6, IL-8, IL-1α, IL-1β).  MHY2233 and resveratrol significantly reduced the percentage of apoptotic cells compared to the control, with MHY2233 showing a slightly stronger effect despite its lower concentration. | MHY2233 is a novel potent SIRT1 activator that can prevent replicative and oxidative stress-induced senescence in EPCs more effectively than resveratrol, suggesting its potential as a therapeutic agent for age-associated cardiovascular diseases. |
| (29) | Li et al. | 2021 | China | In vivo | Natural: Ginsenoside Rg1 (5 and 10 mg/kg), Apocynin (50 mg/kg)  Synthetic: Tempol (50 mg/kg) | Mouse | Liver tissues of senescence-accelerated mouse prone 8 (SAMP8) mice and senescence-accelerated resistant mouse 1 (SAMR1) mice | Reducing oxidative stress by decreasing ROS levels and NOX4 expression  Inhibiting NLRP3 inflammasome activation  Decreasing NF-κB phosphorylation  Decreasing cytokine secretion (IL-1β)  Reducing collagen deposition and fibrosis & Maintaining liver cell morphology | Rg1, apocynin and tempol all reduced ROS production, with Rg1 showing more pronounced effect  Rg1, apocynin and tempol all reduced NOX4 expression in liver tissues of SAMP8 mice  Rg1, apocynin and tempol all decreased expression of NLRP3 inflammasome components (Rg1 showing more reduction), NF-κB phosphorylation, and IL-1β levels  Rg1, apocynin and tempol reduced collagen deposition and liver fibrosis markers (Collagen IV and TGF-β1). The antioxidants maintained normal liver cell morphology in aging mice. | Both natural (Rg1, apocynin) and synthetic (tempol) antioxidants can ameliorate aging-related liver injury and fibrosis by reducing oxidative stress and inflammation. Rg1 appears to have superior effects, suggesting potential advantages of this natural antioxidant for preventing liver aging. |
| (30) | Shen et al. | 2020 | China | In vivo | Natural: Ginsenoside Rg1 (5, 10 mg/kg), Apocynin (50 mg/kg)  Synthetic: Tempol (50 mg/kg) | Mouse | Kidney cells of senescence-accelerated mouse prone 8 (SAMP8) mice and senescence-accelerated resistant mouse 1 (SAMR1) mice | Reducing oxidative stress by decreasing ROS levels and NOX4 expression  Inhibiting NLRP3 inflammasome activation  Decreasing lysosomal compartment and activity (SA-β-gal enzyme activity)  Reducing collagen deposition and fibrosis & Maintaining liver cell morphology by maintaining extracellular matrix components (collagen IV)  Inhibiting extracellular matrix-degrading enzymes (TGF-β1)  Decreasing cytokine secretion (IL-1β) | Rg1, Apocynin, and Tempol all showed anti-senescence effects in the kidneys of SAMP8 mice, as evidenced by reduced senescence-associated β-galactosidase (SA-β-gal) activity, decreased blood urea nitrogen and serum creatinine levels, and ameliorated glomerular fibrosis, with Rg1 (10 mg/kg) showing slightly superior effects  Rg1, Apocynin, and Tempol all reduced ROS production expression in the renal cortex, with Rg1 showing more pronounced effect.  Rg1 (10 mg/kg) showed slightly greater effects in reducing NOX4, p22phox, and p47phox expression compared to Apocynin and Tempol.  Rg1, Apocynin, and Tempol all inhibited NLRP3 inflammasome activation by decreasing expression of NLRP3, cleaved caspase-1, and IL-1β. However, only Rg1 significantly reduced ASC expression as well.  In histological analyses, all three compounds ameliorated glomerular mesangial cell proliferation, matrix deposition, and renal fibrosis. Rg1 (especially at 10 mg/kg dose) showed superior effects in reducing collagen IV and TGF-β1 expression compared to Apocynin and Tempol. | Both natural (Rg1, apocynin) and synthetic (tempol) antioxidants can ameliorate renal fibrosis and delay kidney aging in SAMP8 mice by reducing oxidative stress and inhibiting NLRP3 inflammasome activation, with Rg1 showing superior effects overall |
| (31) | Xu et al. | 2018 | China | In vitro | Natural: Ginsenoside Rg1 (1, 5 and 10 μM)  Synthetic: Tempol (100 μM) | Rat | Primary cultured hippocampal neurons isolated from neonatal Sprague-Dawley rats | Reducing oxidative stress by decreasing ROS levels and NOX2 expression  Inhibiting NLRP1 inflammasome activation  Decreasing lysosomal compartment and activity (SA-β-gal enzyme activity)  Decreasing cytokine secretion (IL-1β, IL-18)  Maintaining cell viability and reducing apoptosis | Rg1 and tempol both reduced ROS production in H2O2-treated hippocampal neurons.  Rg1 (5 and 10 μM) significantly decreased expression of NOX2 and p47phox, while tempol had no significant effect  Rg1 had no significant influence on p22phox expression, while Tempol increased p22phox expression in H2O2-treated neurons  Rg1 (5 and 10 μM) and tempol both inhibited NLRP1 inflammasome activation by decreasing expression of NLRP1, ASC and caspase-1  Rg1 (5 and 10 μM) and tempol reduced senescence markers including SA-β-gal activity and β-galactosidase expression, with Rg1 (5 and 10 μM) showing more pronounced effects  Rg1 and tempol decreased secretion of IL-1β and IL-18  Rg1 and tempol reduced neuronal apoptosis (by decreasing the expression of caspase-3) and increased cell viability in H2O2-treated neurons | Both natural (Rg1) and synthetic (tempol) antioxidants can protect against H2O2-induced senescence and damage in hippocampal neurons by reducing oxidative stress and inhibiting NLRP1 inflammasome activation. Rg1 (5 and 10 μM) showed superior effects in reducing NOX2-mediated ROS production compared to tempol. |
| (32) | Xu et al. | 2019 | China | In vitro | Natural: Apocynin (50 μM)  Synthetic: Tempol (50 μM) | Rat | Primary hippocampal neurons isolated from postnatal Sprague Dawley rats | Reducing oxidative stress by decreasing ROS levels and NOX2 expression  Inhibiting NLRP1 inflammasome activation  Decreasing lysosomal compartment and activity (SA-β-gal enzyme activity)  Decreasing cytokine secretion (IL-1β, IL-18)  Maintaining cell viability and reducing apoptosis | Apocynin and tempol both significantly reduced ROS production in hippocampal neurons cultured for 12 days compared to control, with tempol showing a slightly greater reduction compared to apocynin.  Apocynin and tempol inhibited NLRP1 inflammasome activation by decreasing expression of NLRP1, ASC, caspase-1 and IL-1β in hippocampal neurons cultured for 12 days, with apocynin showing a slightly greater reduction in expression of NLRP1, ASC, caspase-1 compared to tempol but tempol showing a slightly greater reduction in expression of IL-1β compared to apocynin.  Apocynin and tempol reduced senescence markers including SA-β-gal activity and β-galactosidase expression, with tempol showing a slightly greater reduction compared to apocynin.  Apocynin and tempol decreased secretion of IL-1β and IL-18 in the supernatant.  Apocynin and tempol reduced neuronal apoptosis and increased MAP2 expression in neurons cultured for 12 day, with tempol showing a slightly greater reduction (17.52% apoptosis rate) compared to apocynin (18.28% apoptosis rate). Tempol appeared to have a stronger effect in increasing MAP2 levels compared to apocynin. | Both natural (apocynin) and synthetic (tempol) antioxidants can protect against senescence and damage in long-term cultured hippocampal neurons by reducing NOX2-mediated ROS production and inhibiting NLRP1 inflammasome activation. This suggests NOX2-NLRP1 signaling plays an important role in neuronal aging. |
| (33) | Zhu et al. | 2023 | China | In vitro and in vivo | Synthetic: Metformin  Natural: Vitamin E (VE), Mulberry leaf extract (MLE) (10 μg/mL, 50 μg/mL, 100 μg/mL) | Human, Nematode worms, Mouse | In vitro: Human normal hepatocyte L-02 cell line  In vivo: Caenorhabditis elegans (C. elegans) nematode worms, Male ICR mice | Reducing oxidative stress by decreasing ROS levels and increasing antioxidant enzymes (SOD, T-AOC)  Decreasing lipid peroxidation (MDA levels)  Improving mitochondrial function by increasing mitochondrial membrane potential  Decreasing lysosomal compartment and activity (SA-β-gal staining)  Activating AMPK-SIRT1-PGC-1α pathway  Activating Nrf2-Keap1 antioxidant pathway  Decreasing liver dysfunction markers (AST, ALT) | MLE, VE and Metformin all reduced oxidative stress markers (MDA levels) and increased antioxidant defences (SOD activity, T-AOC) in mouse serum and L-02 liver cells. Highest dose (100 μg/mL) of MLE showed similar effects to Metformin and VE.  All three treatments decreased liver dysfunction markers (AST, ALT) and improved mitochondrial membrane potential in liver tissue and L-02 cells, with MLE at highest dose (100 μg/mL) having similar efficacy to Metformin and VE.  In L-02 cells, MLE, Metformin and VE all significantly reduced the proportion of senescent cells as measured by SA-β-gal staining. MLE at the highest dose (100 μg/mL) showed comparable reductions to Metformin and VE.  MLE, Metformin and VE activated the AMPK-SIRT1-PGC-1α and Nrf2-Keap1 signaling pathways to varying degrees in both mouse liver tissue and L-02 cells. Highest dose (100 μg/mL) of MLE showed similar activation levels to Metformin and VE. | MLE, especially at highest dose, showed comparable or superior anti-aging effects to Metformin and VE in liver cells and tissues  Natural antioxidants like MLE may be effective alternatives to synthetic compounds for attenuating cellular senescence and oxidative stress |

1. Bjelica S, Diklic M, Dikic D, Kovacic M, Suboticki T, Mitrovic-Ajtic O, et al. Hydroxyurea-induced senescent peripheral blood mesenchymal stromal cells inhibit bystander cell proliferation of JAK2V617F-positive human erythroleukemia cells. FEBS J. 2019;286(18):3647-63.

2. Briganti S, Wlaschek M, Hinrichs C, Bellei B, Flori E, Treiber N, et al. Small molecular antioxidants effectively protect from PUVA-induced oxidative stress responses underlying fibroblast senescence and photoaging. Free Radic Biol Med. 2008;45(5):636-44.

3. Che H, Ma C, Li H, Yu F, Wei Y, Chen H, et al. Rebalance of the Polyamine Metabolism Suppresses Oxidative Stress and Delays Senescence in Nucleus Pulposus Cells. Oxid Med Cell Longev. 2022;2022:8033353.

4. Chen H, Tu M, Liu S, Wen Y, Chen L. Dendrobine Alleviates Cellular Senescence and Osteoarthritis via the ROS/NF-kappaB Axis. Int J Mol Sci. 2023;24(3).

5. Khor SC, Wan Ngah WZ, Mohd Yusof YA, Abdul Karim N, Makpol S. Tocotrienol-Rich Fraction Ameliorates Antioxidant Defense Mechanisms and Improves Replicative Senescence-Associated Oxidative Stress in Human Myoblasts. Oxid Med Cell Longev. 2017;2017:3868305.

6. Jumnongprakhon P, Pasakawee K, Banjongsinsiri P, Donrung N, Daodee S, Chonpathompikunlert P. The effects of ethanolic extract of Okra fruit, Abelmoschus Esculentus(L.) Moench on cellular senescence in aging neuron. Songklanakarin Journal of Science an Technology (SJST). 2021;43(5):1367-73.

7. Khan SY, Awad EM, Oszwald A, Mayr M, Yin X, Waltenberger B, et al. Premature senescence of endothelial cells upon chronic exposure to TNFalpha can be prevented by N-acetyl cysteine and plumericin. Sci Rep. 2017;7:39501.

8. Khatri R, Krishnan S, Roy S, Chattopadhyay S, Kumar V, Mukhopadhyay A. Reactive Oxygen Species Limit the Ability of Bone Marrow Stromal Cells to Support Hematopoietic Reconstitution in Aging Mice. Stem Cells Dev. 2016;25(12):948-58.

9. Lee YH, Yuk HJ, Park KH, Bae YS. Coumestrol induces senescence through protein kinase CKII inhibition-mediated reactive oxygen species production in human breast cancer and colon cancer cells. Food Chem. 2013;141(1):381-8.

10. Lee YH, Bae YS. Phospholipase D2 downregulation induces cellular senescence through a reactive oxygen species-p53-p21Cip1/WAF1 pathway. FEBS Lett. 2014;588(17):3251-8.

11. Li Y, Zhang W, Chang L, Han Y, Sun L, Gong X, et al. Vitamin C alleviates aging defects in a stem cell model for Werner syndrome. Protein Cell. 2016;7(7):478-88.

12. Moon EY, Oh JM, Kim YH, Ryoo IJ, Yoo ID. Clitocybins, novel isoindolinone free radical scavengers, from mushroom Clitocybe aurantiaca inhibit apoptotic cell death and cellular senescence. Biol Pharm Bull. 2009;32(10):1689-94.

13. Okuni N, Honma Y, Urano T, Tamura K. Romidepsin and tamoxifen cooperatively induce senescence of pancreatic cancer cells through downregulation of FOXM1 expression and induction of reactive oxygen species/lipid peroxidation. Mol Biol Rep. 2022;49(5):3519-29.

14. Pan T, Qian Y, Li T, Zhang Z, He Y, Wang J, et al. Acetyl l-carnitine protects adipose-derived stem cells against serum-starvation: regulation on the network composed of reactive oxygen species, autophagy, apoptosis and senescence. Cytotechnology. 2022;74(1):105-21.

15. Park CH, Kim JW. Effect of advanced glycation end products on oxidative stress and senescence of trabecular meshwork cells. Korean J Ophthalmol. 2012;26(2):123-31.

16. Ren C, Hu C, Wu Y, Li T, Zou A, Yu D, et al. Nicotinamide Mononucleotide Ameliorates Cellular Senescence and Inflammation Caused by Sodium Iodate in RPE. Oxid Med Cell Longev. 2022;2022:5961123.

17. Senthil KK, Gokila VM, Mau JL, Lin CC, Chu FH, Wei CC, et al. A steroid like phytochemical Antcin M is an anti-aging reagent that eliminates hyperglycemia-accelerated premature senescence in dermal fibroblasts by direct activation of Nrf2 and SIRT-1. Oncotarget. 2016;7(39):62836-61.

18. Shiwakoti S, Ko JY, Gong D, Dhakal B, Lee JH, Adhikari R, et al. Effects of polystyrene nanoplastics on endothelium senescence and its underlying mechanism. Environ Int. 2022;164:107248.

19. Wang YC, Lee AS, Lu LS, Ke LY, Chen WY, Dong JW, et al. Human electronegative LDL induces mitochondrial dysfunction and premature senescence of vascular cells in vivo. Aging Cell. 2018;17(4):e12792.

20. Wang Z, Chen Z, Jiang Z, Luo P, Liu L, Huang Y, et al. Cordycepin prevents radiation ulcer by inhibiting cell senescence via NRF2 and AMPK in rodents. Nat Commun. 2019;10(1):2538.

21. Wang CY, Wang J, Cao J, Xu J, Wu RM, Xu XL. Activating PGC-1alpha-mediated signaling cascades in the aorta contributes to the amelioration of vascular senescence and atherosclerosis by 2,3,4',5-tetrahydroxystilbene-2-O-beta-d-glycoside. Phytomedicine. 2022;99:154017.

22. Yang M, Teng S, Ma C, Yu Y, Wang P, Yi C. Ascorbic acid inhibits senescence in mesenchymal stem cells through ROS and AKT/mTOR signaling. Cytotechnology. 2018;70(5):1301-13.

23. Yu M, Kim YJ, Kang DH. Indoxyl sulfate-induced endothelial dysfunction in patients with chronic kidney disease via an induction of oxidative stress. Clin J Am Soc Nephrol. 2011;6(1):30-9.

24. Zhou X, Wang Z, Ni Y, Yu Y, Wang G, Chen L. Suppression effect of N-acetylcysteine on bone loss in ovariectomized mice. Am J Transl Res. 2020;12(3):731-42.

25. Bhayadia R, Schmidt BM, Melk A, Homme M. Senescence-Induced Oxidative Stress Causes Endothelial Dysfunction. J Gerontol A Biol Sci Med Sci. 2016;71(2):161-9.

26. Chen Y, Ding S, Zhang H, Sun Z, Shen X, Sun L, et al. Protective effects of ginsenoside Rg1 on neuronal senescence due to inhibition of NOX2 and NLRP1 inflammasome activation in SAMP8 mice. Journal of Functional Foods. 2020;65.

27. Dai X, Chen Y, Yu Z, Liao C, Liu Z, Chen J, et al. Advanced oxidation protein products induce annulus fibrosus cell senescence through a NOX4-dependent, MAPK-mediated pathway and accelerate intervertebral disc degeneration. PeerJ. 2022;10:e13826.

28. Lamichane S, Baek SH, Kim YJ, Park JH, Dahal Lamichane B, Jang WB, et al. MHY2233 Attenuates Replicative Cellular Senescence in Human Endothelial Progenitor Cells via SIRT1 Signaling. Oxid Med Cell Longev. 2019;2019:6492029.

29. Li Y, Zhang D, Li L, Han Y, Dong X, Yang L, et al. Ginsenoside Rg1 ameliorates aging‑induced liver fibrosis by inhibiting the NOX4/NLRP3 inflammasome in SAMP8 mice. Mol Med Rep. 2021;24(5).

30. Shen X, Dong X, Han Y, Li Y, Ding S, Zhang H, et al. Ginsenoside Rg1 ameliorates glomerular fibrosis during kidney aging by inhibiting NOX4 and NLRP3 inflammasome activation in SAMP8 mice. Int Immunopharmacol. 2020;82:106339.

31. Xu TZ, Shen XY, Sun LL, Chen YL, Zhang BQ, Huang DK, et al. Ginsenoside Rg1 protects against H2O2‑induced neuronal damage due to inhibition of the NLRP1 inflammasome signalling pathway in hippocampal neurons in vitro. Int J Mol Med. 2019;43(2):717-26.

32. Xu T, Sun L, Shen X, Chen Y, Yin Y, Zhang J, et al. NADPH oxidase 2-mediated NLRP1 inflammasome activation involves in neuronal senescence in hippocampal neurons in vitro. Int Immunopharmacol. 2019;69:60-70.

33. Zhu Y, Han Y, Wang W, Liang G, Qi J. Mulberry leaves attenuate D-galactose-induced aging in vivo and in vitro. J Ethnopharmacol. 2023;311:116286.
